# Determinism, well-posedness, and applications of the ultrahyperbolic wave equation in spacekime

**DOI:** 10.1016/j.padiff.2022.100280

**Published:** 2022-02-01

**Authors:** Yuxin Wang, Yueyang Shen, Daxuan Deng, Ivo D. Dinov

**Affiliations:** a Department of Mathematics, University of Michigan, Ann Arbor, MI 48109, USA; b Electrical Computer Engineering Division, University of Michigan, Ann Arbor, MI 48109, USA; c Department of Statistics, University of Michigan, Ann Arbor, MI 48109, USA; d Statistics Online Computational Resource, Department of Health Behavior and Biological Sciences, University of Michigan, Ann Arbor, MI 48109, USA; e Department of Computational Medicine and Bioinformatics, University of Michigan, Ann Arbor, MI 48109, USA

**Keywords:** PDEs, Statistics, Data science, Complex time, Kime, Spacekime analytics

## Abstract

Spatiotemporal dynamics of many natural processes, such as elasticity, heat propagation, sound waves, and fluid flows are often modeled using partial differential equations (PDEs). Certain types of PDEs have closed-form analytical solutions, some permit only numerical solutions, some require appropriate initial and boundary conditions, and others may not have stable, global, or even well-posed solutions. In this paper, we focus on one-specific type of second-order PDE — the ultrahyperbolic wave equation in multiple time dimensions. We demonstrate the wave equation solutions in complex time (kime) and show examples of the Cauchy initial value problem in space-kime.

We extend the classical formulation of the dynamics of the wave equation with respect to positive real longitudinal time. The solutions to the Cauchy boundary value problem in multiple time dimensions are derived in Cartesian, polar, and spherical coordinates. These include both bounded and unbounded spatial domains. Some example solutions are shown in the main text with additional web-based dynamic illustrations of the wave equation solutions in space-kime shown in the appendix. Solving PDEs in complex time has direct connections to data science, where solving under-determined linear modeling problems or specifying the initial conditions on limited spatial dimensions may be insufficient to forecast, classify, or predict a prospective value of a parameter or a statistical model. This approach extends the notion of data observations, anchored at ordered longitudinal events, to complex time, where observables need not follow a strict positive-real structural arrangement, but instead could traverse the entire kime plane.

## 1. Introduction

Many problems tracking the spatiotemporal dynamics of observable processes, such as elasticity, propagation of heat or sound, fluid flows, wave transmission, electrostatic distributions, and electrodynamics, can be mathematically modeled using partial differential equations (PDEs).^1,2^ While there is no general unifying framework for solving all possible PDEs, in many situations, subject to appropriate initial and boundary conditions, their solutions may reliably represent physical processes within specific domains. Most PDEs connect two or more independent variables via an unknown potential function whose partial derivatives with respect to the variables satisfy specific general equations and initial value constraints. To help with explication of potential functions as PDE solutions, the order and complexity of PDEs are used to classify them into different types. The PDE type classification helps with identification of appropriate initial and boundary conditions and the design of effective numerical methods for solving the equations. A general analytic solution of a PDE contains all possible solutions of the equation into a unified analytical framework. Alternatively, when an exact closed-form analytic solution may not be derived, numeric solutions may sometimes provide relevant answers. Examples of techniques commonly used to obtain numeric solutions to PDEs include finite difference or finite elements, gradient discretization, spectral, mesh-free particles, and multigrid methods.^3,4^

This paper is focused on the ultrahyperbolic wave equation in multiple time dimensions. The manuscript is organized as follows. In Section 2, we present the classical formulation of the wave equation in spacetime. The following Section 3 demonstrates the Cartesian coordinate representation of the wave equation in multiple time dimensions, over a bounded spatial domain, and focuses on the special case of complex time (kime).^5–7^ Section 4 derives the solutions of the ultrahyperbolic wave equation over a bounded spatial domain in 2D polar kime coordinates. The wave equation solutions in spherical coordinates, 3D time, and a bounded spatial domain, is presented in Section 5. Several explicit examples are shown in this section. Finally, in Section 6, we derive the solutions of the ultrahyperbolic wave equation for two or more time dimensions and Cartesian coordinates over an unbounded spatial domain. The appendix contains additional examples with dynamic illustrations of the wave equation solutions in space-kime.

A general PDE is defined as an implicit function ℐ of a potential function of multiple space-like (***x***) and time-like (***t***) variables $u=u\left(\underset{x}{\underset{︸}{x_{1},x_{2},\ldots ,x_{n}}},\underset{t}{\underset{︸}{t_{1},t_{2},\ldots ,t_{n}}}\right)$:

(1.1) $$\begin{array}{l} ℐ\left(u\right)=ℐ\left(x,t,u,\frac{\partial u}{\partial x_{1}},\frac{\partial u}{\partial x_{2}},\ldots ,\frac{\partial u}{\partial x_{n}},\frac{\partial u}{\partial t_{1}},\frac{\partial u}{\partial t_{2}},\ldots ,\frac{\partial u}{\partial t_{n}},\ldots ,\right.\\ \;\;\;\;\;\left.\frac{\partial^{2p}u}{\partial x_{1}^{l_{1}}\partial x_{2}^{l_{2}}\cdots \partial x_{n}^{l_{n}}\partial t_{1}^{m_{1}}\partial t_{2}^{m_{2}}\cdots \partial t_{n}^{m_{n}}}\right)=0. \end{array}$$

Many PDE problems also involve specifying sets of constraints (data), *g* = *g* (***x, t***), on the solutions.

The indices *l*_*i*_, $m_{j}\in \mathbb{N}$, $p=\sum_{i=1}^{n}l_{i}=\sum_{j=1}^{n}m_{j}$, and the number of spatial (***x***) and temporal (***t***) variables can be assumed to be the same; otherwise, we can introduce dummy variables with trivial corresponding coefficients to balance the size of the spatial and temporal variables.

The order of the PDE, $q=\max\sum_{i=1}^{n}\left(l_{i}+m_{i}\right)$, is the largest sum of powers of partial derivatives that correspond to non-trivial coefficients in the equation. Linear PDEs represent operators ℐ that depend only linearly on *u* and its derivatives. When the derivatives corresponding to the maximal order only appear in linear terms in the unknown potential function *u* and its derivatives, the PDE is called *quasi-linear*. A PDE is called *homogeneous*, when the equation does not contain terms independent of the unknown potential function, *u*.

The particular type of PDEs that we study in this paper are restricted to linear partial differential equations that can be expressed as:

(1.2) $$\Delta_{x}u=\Delta_{t}u$$

where the Laplacian operators, $\Delta_{x}\equiv \nabla_{x}^{2}$ and $\Delta_{t}\equiv \nabla_{t}^{2}$, represent the unmixed second order partial derivatives of the spatial and temporal variables, respectively. We will solve this equation on domains with relatively simple boundaries (e.g., squares, cylinders, balls).

Classically, linear PDEs with relatively simple boundary conditions are solved using the Fourier transform.^8,9^ The Fourier transform operator ℱ takes a function $f:{\mathbb{R}}^{n}\to \mathbb{C}$, and returns another function, Denoted $\hat{f}:{\mathbb{R}}^{n}\to \mathbb{C}$. The exact form of Fourier transform depends on the specific normalization factor expressed as a constant multiplier ensuring the consistency of the forward and inverse Fourier transformations. In what follows, we adopt the following convention:

(1.3) $$\begin{array}{l} \underset{\text{Fourier transform}}{\underset{︸}{ℱ(f)(\xi )}}\;\;\;\;\;\;\;\;\;\;\;=\hat{f}(\xi ):=\int_{{\mathbb{R}}^{n}}f(x)\cdot e^{-2\pi ix\cdot \xi }dx,\\ \underset{\text{Inverse Fourier transform}}{\underset{︸}{{ℱ}^{-1}(\hat{f})(x)}}=\hat{\hat{f}}(x):=\int_{{\mathbb{R}}^{n}}\hat{f}(\xi )\cdot e^{2\pi ix\cdot \xi }d\xi . \end{array}$$

The power of Fourier transform in solving linear PDEs lies in the fact that ℱ converts differentiation into multiplication; multiplicative equations are in general easier to solve than their differential counterparts. Before we justify the meaning of this statement let us first consider the action of the Fourier transform on a smooth function, $f:{\mathbb{R}}^{n}\to \mathbb{C}$, that decays rapidly at infinity. In this case, we restrict ourselves to the solution space of exponentially decaying functions or Schwartz functions^10^:

(1.4) $$f\in 𝒮\left({\mathbb{R}}^{n}\right)=\left\{\underset{\text{smooth }}{\underset{︸}{f\in C^{\infty }\left({\mathbb{R}}^{n}\right)}}\left|\underset{x\in {\mathbb{R}}^{n}}{\sup}\left|x^{\alpha }\partial^{\beta }f(x)\right|<\infty \right.\text{ for all }\alpha ,\beta \in {\mathbb{N}}^{n}\right\}.$$

For Schwartz functions, integration by parts yields that the Fourier transform of the partial derivative is proportional to the Fourier transform (FT) of the original function. That is, for a given variable index *j*, the FT of the partial derivative $\partial_{j}f:=\frac{\partial }{\partial x_{j}}f$ is:

(1.5) $$\begin{array}{l} ℱ\left(\partial_{j}f\right)(\xi )=\int_{{\mathbb{R}}^{n}}\partial_{j}f(x)\cdot e^{-2\pi ix\cdot \xi }dx=-\int_{{\mathbb{R}}^{n}}f(x)\cdot \partial_{j}e^{-2\pi ix\cdot \xi }dx=\\ \;-\int_{{\mathbb{R}}^{n}}f(x)\cdot \left(-2\pi i\xi_{j}\right)\cdot e^{-2\pi ix\cdot \xi }dx=\left(2\pi i\xi_{j}\right)\cdot \int_{{\mathbb{R}}^{n}}f(x)\cdot e^{-2\pi ix\cdot \xi }dx\\ \;=\left(2\pi i\xi_{j}\right)\cdot ℱ(f)(\xi ). \end{array}$$

Thus, to solve a PDE of the form

(1.6) $$\sum_{j=1}^{d}\alpha_{j}\partial_{j}f(x)=g(x),$$

we can apply the Fourier transform to both sides of the equation:

(1.7) $$\hat{g}(\xi )=ℱ\left(\sum_{j=1}^{d}\alpha_{j}\partial_{j}f(x)\right)=\sum_{j=1}^{d}\alpha_{j}\cdot ℱ\left(\partial_{j}f(x)\right)=\;\sum_{j=1}^{d}\alpha_{j}\cdot \left[\left(2\pi i\xi_{j}\right)\cdot ℱ(f)(\xi )\right]=\left[\sum_{j=1}^{d}\alpha_{j}\cdot \left(2\pi i\xi_{j}\right)\right]\cdot ℱ(f)(\xi )$$

The solution of this equation is:

(1.8) $$ℱ(f)(\xi )=\frac{\hat{g}(\xi )}{\sum_{j=1}^{d}\alpha_{j}\cdot \left(2\pi i\xi_{j}\right)}.$$

Therefore, given the PDE right-hand-side, *g* (*x*), we can compute its Fourier transform, $\hat{g}(\xi )$, the Fourier transform of the corresponding potential function, $ℱ(f)=\hat{f}$, and explicitly derive the PDE solution via the inverse Fourier transform (IFT):

(1.9) $$f(x)={ℱ}^{-1}(\hat{f}(\xi ))(x)=\int_{{\mathbb{R}}^{n}}\hat{f}(\xi )\cdot e^{2\pi ix\cdot \xi }d\xi .$$

Historically, PDEs have been classified as ‘‘*elliptic*’’, ‘‘*parabolic*’’, or ‘‘*hyperbolic*’’.^9,11,12^ Naturally, most of the fundamental PDEs, namely the Laplace equation, heat equation, and wave equation, fall into these types of categories. Contrasting the differences between separate partial differential equations may be accomplished by cataloging and classifying their types. Below we will characterize these three types of PDEs.

Denoting *lower order terms* by ‘‘l.o.t.’’, a (second order) *elliptic equation* is of the form:

(1.10) $$Au_{xx}+2Bu_{xy}+Cu_{yy}+\underset{\text{1}.\text{o}.\text{t}.}{\underset{︸}{Du_{x}+Eu_{y}+Fu+G}}=0$$

where $B^{2}-AC<0$. The Laplace equation, *∆u* = 0, represents a prototypical example of an elliptic partial differential equation.

It is known that qualitatively, solutions to the heat equation have a gain of two derivatives.^13^ For example, harmonic functions are automatically infinitely smooth. And many elliptic equations have similar properties, although the estimates may be harder to obtain in non-linear circumstances.

Elliptic equation problems are typically formulated with boundary constraints, rather than initial conditions, e.g., the solution to the equation *∆u* = 0 may be constrained by a restriction of the form ${\left.u\right|}_{\partial \Omega }=f(x)$ for some given function *f*(*x*). In general, elliptic PDEs are actually ill-posed for general initial conditions. In general, elliptic equations come with boundary value conditions, rather than initial value conditions. That is, the restricting condition on *∆u* = 0 is usually of the form ${\left.u\right|}_{\partial \Omega }$. This is in contrast to initial conditions, as discussed below.

*Boundary conditions* usually restrict the potential function solution on a given boundary (submanifold) of the (spatial) domain. *Initial conditions* impose constraints on the solution at the initial time *t* = 0. For instance, the initial conditions for the heat equation *u*_*t*_ = *u*_*xx*_, for *t* ≥ 0 and 0 ≤ *x* ≤ 1, may be stated as *u* (*x,* 0) = *ℎ* (*x*). Whereas, the boundary constraints may be formulated as *u* (0*, t*) = *f* (*t*) *, u* (1*, t*) = *g* (*t*) (Dirichlet boundary conditions) or as *u_x_* (0*, t*) = *f* (*t*) *, u*_*x*_ (0*, t*) = *g* (*t*) (Neumann boundary conditions), or by specifying periodic boundary conditions like *u* (1*, t*) = *u* (0*, t*).

In principle, the boundary and initial conditions could be more complex, involve higher order derivatives, require the solutions to be in various functional spaces, or demand certain potential function differentiability or integrability. For instance, Robin boundary conditions represent linear combinations of Neumann and Dirichlet terms.

To solve the PDE over a region *Ω* with a boundary ∂Ω, let us denote by **𝐧** the normal direction. Then, the *normal derivative* of the potential function at the boundary ∂Ω is

(1.11) $$\frac{\partial u}{\partial n}=\nabla u(x)\cdot n=\nabla_{n}u(x)=\frac{\partial u}{\partial x}\cdot n.$$

Examples of generic boundary conditions include:

Robin conditions:

(1.12) $$\begin{array}{l} {\left.g(x)=\left(\alpha (x)u+\beta (x)\frac{\partial u}{\partial n}\right)\right|}_{\partial \Omega },\\ \alpha (x),\beta (x):\partial \Omega \to \mathbb{R},\\ \alpha (x)\geq 0,\beta (x)\geq 0,\alpha (x)+\beta (x)>0\;\text{on }\partial \Omega ,\\ \text{Dirichlet conditions}:\beta (x)=0,\forall x,\\ \text{Neumann}:\alpha (x)=0,\forall x,\\ \text{Robin}:\alpha (x),\beta (x)\neq 0,\forall x. \end{array}$$

In general, a *well-posed* mathematical problem is one that guarantees the following three properties:

1. A solution exists (at least locally in a short time range, if not globally),
2. The solution is unique (in some measure space), and
3. The solution depends continuously on the initial data. That is, the solution to a constrained problem with initial condition *u* (*x,* 0) = *ℎ* (*x*) will only change proportionally to a small perturbation of *ℎ* (*x*).

Conversely, a mathematical problem is *ill-posed* if any of these three conditions are not satisfied. Note that well-posedness and ill-posedness depend on the functional space. A problem that is well-posed on one functional space could very well be ill-posed in another (weaker) functional space. An example is the heat equation; if we require that *u* is bounded as *x* → ±∞, then the problem is well-posed, however, without this additional constraint, the heat equation solution is no longer unique, and hence, the problem is not well-posed.

Let us now review the (second order) *parabolic equation*, which has the form:

(1.13) $$Au_{xx}+2Bu_{xy}+Cu_{yy}+\text{ l}.\text{o}.\text{t}.=0,$$

where $B^{2}-AC=0$. A prototypical example of a parabolic PDE is the heat equation *u*_*t*_ = *u*_*xx*_.

To realize the heat equation as a parabolic PDE, we can consider the second variable *y* as time *𝑡* and take *A* = 1*,B* = 0*,C* = 0. The heat equation is known for its instant smoothing behavior. That is, given any (rough) initial data *u*_0_ (*x*), the solution to the heat equation *u* (*t, x*) is infinitely smooth in *x*. Moreover, locality is not preserved and any perturbation to the initial condition can instantly propagate to any *x* location.

Parabolic equations can be solved with both initial and boundary conditions. The initial condition can be specified in the form ${\left.u\right|}_{t=0}=u_{0}\left(x\right)$ for some given function *u*_0_ (*x*), and various boundary conditions such as ${\left.u\right|}_{\partial \Omega }=f(x)$, or ${\left.u_{x}\right|}_{\partial \Omega }=f(x)$ can also be imposed. More complicated boundary conditions can also be imposed. For instance, periodic boundary condition of the form ${\left.u\right|}_{x=0}={\left.u\right|}_{x=1}$, or Robin boundary condition of the form $a(x)u(x)+b(x)\nabla_{n}u=g(x)$ on *∂Ω*.

Note that adding non-linear lower order terms may change the structure of the equation drastically. For example, *𝑢*_*𝑡*_ = *𝑢*_*xx*_ + *𝑢*_*x*_ is still a parabolic equation, but *u*_*t*_ = *u*_*xx*_ + (*u*_*x*_)^2^ is not. The reason is that multiplying both sides of *u*_*t*_ = *u*_*xx*_ + *u*_*x*_ by *u* and integrating against *x* yields:

(1.14) $$\begin{array}{l} \int u\cdot u_{t}=\int u\cdot u_{xx}+\int u\cdot u_{x},\\ \frac{1}{2}\partial_{t}\int {\left(u\right)}^{2}=\underset{\begin{array}{c} \text{ u decays fast }\\ \text{ at infinity } \end{array}}{\underset{︸}{\int_{\partial \Omega }u\cdot u_{x}}}-\int {\left(u_{x}\right)}^{2}-\int u\cdot u_{x}\\ \;\;\;\;\;=-\int {\left(u_{x}\right)}^{2}-\int u\cdot u_{x}. \end{array}$$

Since $\int u\cdot u_{x}=-\int u\cdot u_{x}$, we conclude that $\int u\cdot u_{x}=0$. Thus, $\frac{1}{2}\partial_{t}\int {\left(u\right)}^{2}=-\int {\left(u_{x}\right)}^{2}\leq 0$ and $\|u{\|}^{2}$ is non-increasing. This suggests stability of the solution to the PDE with lower-order linear terms. However, including additive quadratic terms makes the parabolic heat equation unstable. For instance, consider $u_{t}=u_{xx}+{\left(u_{x}\right)}^{2}$. Again, we can multiply both sides by *u* and integrate with respect to *x*:

(1.15) $$\frac{1}{2}\partial_{t}\int {\left(u\right)}^{2}=-\int {\left(u_{x}\right)}^{2}+\int u\cdot {\left(u_{x}\right)}^{2},$$

where $\int u\cdot {\left(u_{x}\right)}^{2}$ is usually non-zero and large (e.g., for discontinuous initial data). Hence, modification of the heat equation by adding a non-linear lower-order term yields instability of the solution.

Finally, a (second order) *hyperbolic equation* is of the form:

(1.16) $$Au_{xx}+2Bu_{xy}+Cu_{yy}+\text{ l}.\text{o}.\text{t}.=0,$$

where $B^{2}-AC>0$. The wave equation, *u*_*tt*_ = *u*_*xx*_, represents a classical example of a hyperbolic PDE. There is usually *no gain of regularity* for the wave equation. Furthermore, wave equations are subject to finite speed of propagation, as will be discussed below. Similar to parabolic equations, *hyperbolic equations* also come with various kinds of initial and boundary conditions. No gain of regularity implies that the solution to the wave equation is as smooth, or as rough, as its initial condition. This is in contrast to the heat equation, which has a gain of regularity because the initial condition could be very rough (e.g. discontinuous), whereas the solution is always smooth.

Table 1 summarizes some of the main partial differential equations and their classification types. Note that certain types of PDEs may change from one classification type to another depending on the conditions. For example, based on their linearized form, the Navier–Stokes equations span the three PDE types reflecting their diverse characteristic behavior.^14^ Depending on the governing parameters, one behavior can be dominant, e.g., the Navier–Stokes equations may be parabolic (diffusion dominated models of unsteady viscous flows including a nontrivial diffusion term), or elliptic (for steady viscous flows), or even hyperbolic (when advection is dominant).

Note that the exemplary initial and boundary conditions discussed above are not the only possible conditions that can be imposed. More exotic conditions are also possible and sometimes commonly used in the PDE field.^8,14^

For example, ultrahyperbolic PDEs represent another kind of equations that are closely related to the hyperbolic equations. In classical spacetime, the wave equation takes the form *u*_*tt*_ = *∆*_*x*_*u* where the temporal variable *t* is 1-dimensional (positive real) and the spatial variable *x* may be multi-dimensional. The more general ultrahyperbolic wave equation is of the same form as the classical wave equation, *∆*_*t*_*u* = *∆*_*x*_*u*, however, it includes two or more temporal (time-like) dimensions. The focus of this manuscript is on these ultrahyperbolic wave equations. The behavior of the ultrahyperbolic equation solutions are drastically different from the behavior of their hyperbolic counterparts, the wave equation with univariate time.

This article examines the determinism, well-posedness, and applications of ultrahyperbolic partial differential equations. The specific goals of the paper include explicit identification of the initial and boundary value conditions for the ultrahyperbolic wave equation, the analytic formulation of the solutions in different coordinate systems, and a demonstration of the polar coordinates (complex time) dynamics of the potential function solutions. A number of interesting data science applications emerge from the extension of the dynamics from standard (positive real) time to complex time (kime). Examples of such applications using heterogeneous longitudinal neuroimaging, economics, and clinical data are presented elsewhere.^7^

We explore the existence, uniqueness and stability of the solutions of the wave equation in multiple time dimensions, as a special case of ultrahyperbolic partial differential equations. The manuscript is organized as follows. In the second section, we present the Cartesian coordinate representation of the wave equation in multiple time dimensions and focus on the special case of complex time (kime).^5–7^ In section three, we present the solutions of the ultrahyperbolic wave equation over a bounded spatial domain in 2D polar kime coordinates. In sections four and five, we extend the kime solutions over a bounded spatial domain to 2D polar and 3D spherical coordinates, respectively. Section six presents the Cartesian coordinate solutions for an unbounded spatial domain. Finally, the last section presents a discussion and offers some open problems. The supplementary appendix shows some applications and provides references to online dynamic animations depicting the wave equation solutions in higher time dimensions.

## 2. Spacetime wave equation

The spacetime wave equation, *u*_*tt*_ = *∆*_*x*_*u*, where t∈ℝ and $x=\left(x_{1},\ldots ,x_{n}\right)\in D_{x}\subset {\mathbb{R}}^{n}$, is a prototypical example for hyperbolic Partial Differential Equations (PDE). It models the dynamics of waves (as its name suggests), and more generally propagation of information. The significance of wave equation lies in the following properties:

- *Finite speed of propagation*.

Indeed, the solution to an initial value problem

(2.1) $$u(t=0)=u_{0}(x)\;u_{t}(t=0)=u_{1}(x)$$

is given by Kirchhoff’s formula, which says that the solution is

(2.2) $$\begin{array}{l} u(x,t)=\frac{1}{\left|\partial B(x,t)\right|}\int_{\partial B(x,t)}t\times u_{1}(y)dy+\frac{1}{\left|\partial B(x,t)\right|}\\ \times \int_{\partial B(x,t)}u_{0}(y)+\nabla u_{0}(y)\cdot (y-x)dS(y). \end{array}$$

This solution formula establishes the celebrated finite speed of wave propagation: waves, and information in general, could only travel at a finite speed, so events that happened far away will not be felt by an observer until sometime later. This feature is distinct for hyperbolic equations; for parabolic equations, e.g., the heat equation, any minor disturbance can be felt instantly by an observer arbitrarily far away.

- Conservation of energy

Remarkably, energy is preserved in traveling wave: if we set $E=\frac{1}{2}\int_{{\mathbb{R}}^{n}}{\left|u_{t}\right|}^{2}+{\left|\nabla_{x}u\right|}^{2}dx$, then

(2.3) $$\frac{d}{dt}E=\int_{{\mathbb{R}}^{n}}u_{t}\cdot u_{tt}dx+\int_{{\mathbb{R}}^{n}}\langle \nabla_{x}u,\nabla_{x}u_{t}\rangle \;dx\;\;\;=\int_{{\mathbb{R}}^{n}}u_{t}\cdot \overset{u_{tt}=\Delta_{x}u}{\overset{︷}{\Delta_{x}u}}dx-\int_{{\mathbb{R}}^{n}}\Delta_{x}u\cdot u_{t}dx=0$$

where in the second equality, we integrated by part and used the assumption that if energy is finite, then *u* has to vanish at infinity.

- Symmetry
  Another property of the wave equation is that the solution is invariant under the transformation t→−t and x→O⋅x where *O* is an orthogonal matrix, which shows the symmetry of the equation.
- Solution in frequency space
  We can a take special Fourier transform of the equation *u*_*tt*_−*∆*_*x*_*u* = 0 to obtain the equation on the frequency side:${\hat{u}}_{tt}+|\xi {|}^{2}\hat{u}(\xi )=0$. Solving the ODE in *t*, we obtain

(2.4) $$\hat{u}(\xi ,t)=C_{1}\cdot \cos(|\xi |t)+C_{2}\cdot \sin(|\xi |t).$$

Combining with the initial conditions, we have

(2.5) $$\hat{u}(\xi ,t)={\hat{u}}_{0}(\xi )\cdot \cos(|\xi |t)+\frac{{\hat{u}}_{1}(\xi )}{\left|\xi \right|}\cdot \sin(|\xi |t).$$

This shows that the initial value problem is globally well-posed.

For nonlinear hyperbolic PDEs, many properties can be easily generalized. For instance, the proof of local well-posedness of the Euler equation utilizes a priori estimate that is similar to conservation of energy. In this sense, the spacetime wave equation plays a crucial role in understanding hyperbolic PDEs.

However, as we generalize to multiple time dimensions, not all properties are maintained. For instance, while we can establish a similar conservation of ‘‘energy’’, the energy is no longer positive. Thus, extra care must be taken in solving the spacekime wave equation, *aka* the ultrahyperbolic wave equation. Nonetheless, we shall use methods motivated by the spacetime wave equation to investigate the wave equation in spacekime.

## 3. Cartesian coordinate and bounded spatial domain problem

To emphasize the direct application to spacekime analytics, we will denote the time-like variables by kappa, i.e.,t≡κ. In its most general form, the extension of the wave equation in higher dimensions is a natural generalization of its spacetime analogue and still represents a second-order linear PDE:

(3.1) $$\begin{array}{l} \underset{\text{spatial Laplacian }}{\underset{︸}{\Delta_{x}u(x,\kappa )}}=\underset{\text{temporal Laplacian }}{\underset{︸}{\Delta_{\kappa }u(x,\kappa )}},\\ \Delta_{x}u=\sum_{i=1}^{d_{s}}\partial_{x_{i}}^{2}u,\\ \Delta_{\kappa }u=\sum_{i=1}^{d_{t}}\partial_{\kappa_{i}}^{2}u, \end{array}$$

where $x=\left(x_{1},x_{2},\ldots ,x_{d_{s}}\right)\in {\mathbb{R}}^{d_{s}}$ and $\kappa =\left(\kappa_{1},\kappa_{2},\ldots ,\kappa_{d_{t}}\right)\in {\mathbb{R}}^{d_{t}}$ are the Cartesian coordinates in the *d*_*s*_ space and *d*_*t*_ time dimensions. Of course, there may also be different weights, ${\left\{\alpha_{i}\right\}}_{i=1}^{d_{s}}$ and ${\left\{\beta_{j}\right\}}_{j=1}^{d_{t}}$, that can be introduced with each space or time dimension:

(3.2) $$\underset{\Delta_{x}^{\text{α}}u(x,\kappa )}{\underset{︸}{\sum_{i=1}^{d_{s}}\alpha_{i}\partial_{x_{i}}^{2}}}u=\underset{\Delta_{x}^{\text{β}}u(x,\kappa )}{\underset{︸}{\sum_{j=1}^{d_{t}}\beta_{j}\partial_{\kappa_{j}}^{2}u}}.$$

The special case involving the smallest flat 5D spacekime manifold has *d*_*s*_ = 3 space-like (spatial) and *d*_*t*_ = 2 time-like (temporal) variables, $x=\left(x_{1},x_{2},x_{3}\right)\in {\mathbb{R}}^{3}$ and $\kappa =\left(\kappa_{1},\kappa_{2}\right)\in \mathbb{C}≅{\mathbb{R}}^{2}$, respectively.

To derive solutions to the wave equation in spacekime, or in higher dimensions, and explore their existence, validity, and stability, let us start with a simpler problem of functions defined with periodic boundary conditions. The *d*_*s*_-dimensional spatial hypercube is

(3.3) $$x=\left(x_{1},x_{2},\ldots ,x_{d_{s}}\right)\in D_{s}\equiv \underset{d_{s}}{\underset{︸}{\left[-1,1]\;\times \;[-1,1]\;\times \cdots \;\times \;[-1,1\right]}}\equiv {\left[-1,1\right]}^{d_{s}}\subset {\mathbb{R}}^{d_{s}}$$

and *d*_*t*_-dimensional temporal hypercube is

(3.4) $$t=\left(\kappa_{1},\kappa_{2},\ldots ,\kappa_{d_{t}}\right)\in D_{t}\equiv \underset{d_{t}}{\underset{︸}{\left[-1,1]\;\times \;[-1,1]\;\times \cdots \;\times \;[-1,1\right]}}\equiv {\left[-1,1\right]}^{d_{t}}\subset {\mathbb{R}}^{d_{t}}.$$

Let $\eta ={\left(\eta_{1},\eta_{2},\ldots ,\eta_{d_{t}}\right)}^{\prime }$ and $\text{ξ}={\left(\xi_{1},\xi_{2},\ldots ,\xi_{d_{s}}\right)}^{\prime }$ represent respectively the frequency vectors of *integers* corresponding to the temporal and spatial frequencies of the Fourier-transformed periodic solution of the wave equation. In general, when dealing with non-periodic functions, the spatial and temporal frequencies may be real or complex numbers, but for our periodic boundary condition case, the frequencies are integers. Assuming that the solution is twice continuously differentiable, i.e., $u\in C^{2}\left(D_{t}\times D_{s}\right)\subseteq L^{2}$, we will use the Fourier transform,$ℱ:L^{2}\to L^{2}$:

(3.5) $$\begin{array}{l} U(\eta ,\text{ξ})=ℱ(u)(\eta ,\text{ξ})=\int_{D_{s},D_{t}}u(x,\kappa )\times e^{-2\pi i\langle \eta ,\kappa \rangle }\times e^{-2\pi i\langle \text{ξ},x\rangle }dxd\kappa ,\\ u(x,\kappa )={ℱ}^{-1}(U)(x,\kappa )=\int_{D_{\eta },D_{\text{ξ}}}U(\eta ,\text{ξ})\times e^{2\pi i\langle \eta ,\kappa \rangle }\times e^{2\pi i\langle \text{ξ},x\rangle }d\eta d\text{ξ}. \end{array}$$

In general, there is no direct algebraic relation between an arbitrary function *f* = *f* (***y***) and its Laplacian $\Delta f=\sum_{i=1}^{d}\partial_{y_{i}}^{2}f$. However, in the special case of a planar wave, $f(y)=e^{2\pi i\langle y,\text{ξ}\rangle }$, the Laplacian has the following interesting property:

(3.6) $$\Delta f=\Delta e^{2\pi i\langle y,\text{ξ}\rangle }=\underset{\lambda }{\underset{︸}{-4\pi^{2}|\xi {|}^{2}}}\times \underset{f}{\underset{︸}{e^{2\pi i\langle y,\text{ξ}\rangle }}}.$$

In other words, plane waves, $f(y)=e^{2\pi i\langle y,\text{ξ}\rangle }$, are *eigenfunctions* of the Laplacian operator, i.e., *∆f* = *λf*, corresponding to the *eigenvalue $\lambda =-4\pi^{2}|\xi {|}^{2}$*. Plane waves are base functions that allow us to represent any *L*^2^ function as a superposition of plane waves, potentially infinitely many plane waves. As the Laplacian is a linear operator, any periodic square-integrable function with a smooth second derivative (necessary to justify integration by parts)$u(y):{\left[-1,1\right]}^{d}\to \mathbb{C}$, including *∆u* (*y*), can be expressed in terms of its Fourier transform,U(ξ)=ℱ(u)(ξ):

(3.7) $$\begin{array}{l} \Delta_{y}u(y)=\Delta u(y)=\Delta \left(\underset{u(y)={ℱ}^{-1}(\text{U})(y)}{\underset{︸}{\int_{D}\text{U}(\text{ξ})\times e^{2\pi i\langle y,\text{ξ}\rangle }d\text{ξ}}}\right)=\int_{D}U(\xi )\Delta \left(e^{2\pi i\langle y,\text{ξ}\rangle }\right)d\text{ξ}=\\ \int_{D}\text{U}(\xi )\underset{\Delta \left(e^{2\pi i\langle y,\text{ξ}\rangle }\right)}{\underset{︸}{\left[\left(-4\pi^{2}|\text{ξ}{|}^{2}\right)e^{2\pi i\langle y,\text{ξ}\rangle }\right]}}d\text{ξ}=\int_{D}\left[\left(-4\pi^{2}|\text{ξ}{|}^{2}\right)\times \text{U}(\text{ξ})\right]e^{2\pi i\langle y,\text{ξ}\rangle }d\text{ξ}. \end{array}$$

Since the integrant $\left(U(\text{ξ})\Delta \left(e^{2\pi i\langle y,\text{ξ}\rangle }\right)\right)$ is in *L*^1^, we can interchange integral and derivative operators in the equation above.

The Fourier transform representation of the Laplacian is:

(3.8) $$\begin{array}{l} \underset{f(y)}{\underset{︸}{\Delta u(y)}}={ℱ}^{-1}\left(\underset{ℱ(f)}{\underset{︸}{\text{F}}}\right)\left(y\right)=\int_{D}\underset{ℱ(f)(\xi )}{\underset{︸}{ℱ(\Delta u)(\text{ξ})}}\times e^{2\pi i\langle y,\text{ξ}\rangle }d\text{ξ}\\ \;\;\;=\int_{D}\underset{ℱ(\Delta u)(\xi )}{\underset{︸}{\left(-4\pi^{2}|\text{ξ}{|}^{2}\right)\times U(\text{ξ})}}e^{2\pi i\langle y,\text{ξ}\rangle }d\text{ξ}. \end{array}$$

We can show that (periodic) potential functions *u* of this type

(3.9) $$u(x,\kappa )=e^{2\pi i\langle \eta ,\kappa \rangle }\times e^{2\pi i\langle x,\text{ξ}\rangle },\;\text{subject to }|\eta {|}^{2}\equiv |\text{ξ}{|}^{2},$$

solve the general wave equation $\Delta_{x}u(x,\kappa )=\Delta_{\kappa }u(x,\kappa )$. Let $u(x,\kappa )=e^{2\pi i\langle \eta ,\kappa \rangle }\times e^{2\pi i\langle x,\text{ξ}\rangle }\in C^{2}\left(D_{t}\times D_{s}\right)$ and integrate by parts the Fourier transform of its *temporal* partial derivatives:

(3.10) $$\begin{array}{l} ℱ\left(\partial_{\kappa_{j}}u\right)(\eta ,\text{ξ})=\int_{D_{s},D_{t}}\partial_{\kappa_{j}}u(x,\kappa )\times e^{-2\pi i\langle \eta ,\kappa \rangle }\times e^{-2\pi i\langle \text{ξ},x\rangle }dxd\kappa =\\ \int_{D_{s}}\int_{D_{t}}\partial_{\kappa_{j}}u(x,\kappa )\times e^{-2\pi i\langle \eta ,\kappa \rangle }\times e^{-2\pi i\langle \text{ξ},x\rangle }dxd\kappa =\\ \int_{D_{s}}\left[\underset{0,\;\text{periodic}\;\text{function}\;\text{on}\;\kappa \in {\text{D}}_{t}\equiv {\left[-1,1\right]}^{d_{t}}}{\underset{︸}{\int_{\partial D_{t}}u\left(x,\kappa \right)\times e^{-2\pi i\langle \eta ,\kappa \rangle }\times e^{-2\pi i\langle \text{ξ},x\rangle }d\kappa }}\right.\\ \left.-\int_{D_{t}}u(x,\kappa )\times \partial_{\kappa_{j}}e^{-2\pi i\langle \eta ,\kappa \rangle }\times e^{-2\pi i\langle \text{ξ},x\rangle }d\kappa \right]dx=\\ \int_{D_{s}}\left[-\int_{D_{t}}\left(-2\pi i\eta_{j}\right)u(x,\kappa )\times e^{-2\pi i\langle \eta ,\kappa \rangle }\times e^{-2\pi i\langle \text{ξ},x\rangle }d\kappa \right]dx=\\ 2\pi i\eta_{j}\int_{D_{s},D_{t}}u(x,\kappa )e^{-2\pi i\langle \eta ,\kappa \rangle }e^{-2\pi i\langle \text{ξ},x\rangle }dxd\kappa =2\pi i\eta_{j}ℱ(u)(\eta ,\text{ξ}),\\ \forall 1\leq j\leq d_{t}. \end{array}$$

Similarly, the Fourier transform of the *spatial* partial derivatives:

(3.11) $$ℱ\left(\partial_{x_{l}}u\right)(\eta ,\text{ξ})=2\pi i\xi_{l}ℱ(u)(\eta ,\text{ξ}),\;\forall 1\leq l\leq d_{s}.$$

Therefore, $\underset{\hat{\Delta u}}{\underset{︸}{ℱ(\Delta u)}}(\text{ξ})=\underset{\text{multiplier }}{\underset{︸}{\left(-4\pi^{2}|\text{ξ}{|}^{2}\right)}}\times \underset{U=\hat{u}}{\underset{︸}{ℱ(u)}}(\text{ξ})$ and hence, the Laplacian operator can be considered as a Fourier multiplier operator. That is, the Fourier transform of the Laplacian (*∆u*) at a frequency ξ is given by the Fourier transform of the original function (*u*) evaluated at the same frequency, multiplied by the value of the multiplier at that frequency,$-4\pi^{2}|\xi {|}^{2}$.

Let us compute the Fourier transform of the second partial derivatives using $\partial_{x_{l}}^{2}u=\partial_{x_{l}}\left(\partial_{x_{l}}\right)(u)$ and $\partial_{\kappa_{j}}^{2}u=\partial_{\kappa_{j}}\left(\partial_{\kappa_{j}}\right)(u)$:

$$\begin{array}{l} ℱ\left(\partial_{\kappa_{j}}^{2}u\right)(\eta ,\text{ξ})=2\pi i\eta_{j}ℱ\left(\partial_{\kappa_{j}}u\right)(\eta ,\text{ξ})\\ \;\;\;\;\;\;={\left(2\pi i\eta_{j}\right)}^{2}ℱ(u)(\eta ,\text{ξ})=-{\left(2\pi \eta_{j}\right)}^{2}ℱ(u)(\eta ,\text{ξ}),\forall 1\leq j\leq d_{t}, \end{array}$$

(3.12) $$\begin{array}{l} ℱ\left(\partial_{x_{l}}^{2}u\right)(\eta ,\text{ξ})=2\pi i\xi_{l}ℱ\left(\partial_{x_{l}}u\right)(\eta ,\text{ξ})={\left(2\pi i\xi_{l}\right)}^{2}ℱ(u)(\eta ,\text{ξ})\\ \;\;\;\;\;\;=-{\left(2\pi \xi_{l}\right)}^{2}ℱ(u)(\eta ,\text{ξ}),\forall 1\leq l\leq d_{s}. \end{array}$$

As the spatial and temporal Fourier transforms are linear:

(3.13) $$\begin{array}{l} ℱ\left(\Delta_{x}u\right)(\eta ,\text{ξ})=ℱ\left(\sum_{l=1}^{d_{s}}\partial_{x_{l}}^{2}u\right)(\eta ,\text{ξ})=\sum_{l=1}^{d_{s}}ℱ\left(\partial_{x_{l}}^{2}u\right)(\eta ,\text{ξ})\\ \;\;\;\;\;\;=-4\pi^{2}|\text{ξ}{|}^{2}ℱ(u)(\eta ,\text{ξ}),\\ ℱ\left(\Delta_{\kappa }u\right)(\eta ,\text{ξ})=ℱ\left(\sum_{j=1}^{d_{t}}\partial_{\kappa_{j}}^{2}u\right)(\eta ,\text{ξ})=\sum_{j=1}^{d_{t}}ℱ\left(\partial_{\kappa_{j}}^{2}u\right)(\eta ,\text{ξ})\\ \;\;\;\;\;\;=-4\pi^{2}|\eta {|}^{2}ℱ(u)(\eta ,\text{ξ}). \end{array}$$

Applying the Fourier transform to the wave equation $\Delta_{x}u(x,\kappa )=\Delta_{\kappa }u(x,\kappa )$ yields:

(3.14) $$\begin{array}{l} -4\pi^{2}|\text{ξ}{|}^{2}ℱ(u)(\eta ,\text{ξ})=ℱ\left(\Delta_{x}u\right)(\eta ,\text{ξ})\\ \;\;\;\;\;\;\;\;\;\;\equiv ℱ\left(\Delta_{\kappa }u\right)(\eta ,\text{ξ})=-4\pi^{2}|\eta {|}^{2}ℱ(u)(\eta ,\text{ξ}). \end{array}$$

This suggests a necessary and sufficient condition $|\text{ξ}{|}^{2}=|\eta {|}^{2}$ for the relation between the integer spatial (ξ) and integer temporal (η) frequencies that guarantee the potential function $u(x,\text{κ})=e^{2\pi i\langle \text{η},\text{κ}\rangle }\times e^{2\pi i\langle x,\xi \rangle }$ represents a wave equation solution.

Since the wave equation is a linear PDE, any finite linear combination of *M* such basic potential functions will also represent a (composite) solution:

(3.15) $$u(x,\kappa )=\underset{\left\{{\text{ξ}}_{m},\eta_{m}\text{ s}.\text{t}.{\left|{\text{ξ}}_{m}\right|}^{2}=\;{\left|\eta_{m}\right|}^{2}\right\}}{\sum_{m=1}^{M}\;}C_{m}\times e^{2\pi i\langle \eta_{m},\kappa \rangle }\times e^{2\pi i\langle x,{\text{ξ}}_{m}\rangle }.$$

In spacekime, using polar coordinate representation of kime, the simple (*M* = 1) separable solutions of the wave equation can be expressed via:

(3.16) $$\begin{array}{c} \kappa =te^{i\theta }=t(\cos\theta +i\sin\theta )=\underset{\left(t\cos\theta \right)}{\underset{︸}{\kappa_{1}}}+i\underset{\kappa_{2}}{\underset{︸}{\left(t\sin\theta \right)}},\\ u(x,\kappa )=e^{2\pi i\langle \eta ,\kappa \rangle }\times e^{2\pi i\langle x,\text{ξ}\rangle }=e^{2\pi i\sum_{j=1}^{d_{t}=2}\kappa_{j}\eta_{j}}\times e^{2\pi i\sum_{l=1}^{d_{s}=3}x_{l}\xi_{l}}=\\ e^{2\pi i\left(\eta_{1}t\cos\theta +\eta_{2}t\sin\theta \right)}\times e^{2\pi i\left(x_{1}\xi_{1}+x_{2}\xi_{2}+x_{3}\xi_{3}\right)}. \end{array}$$

**Fig. 1.**
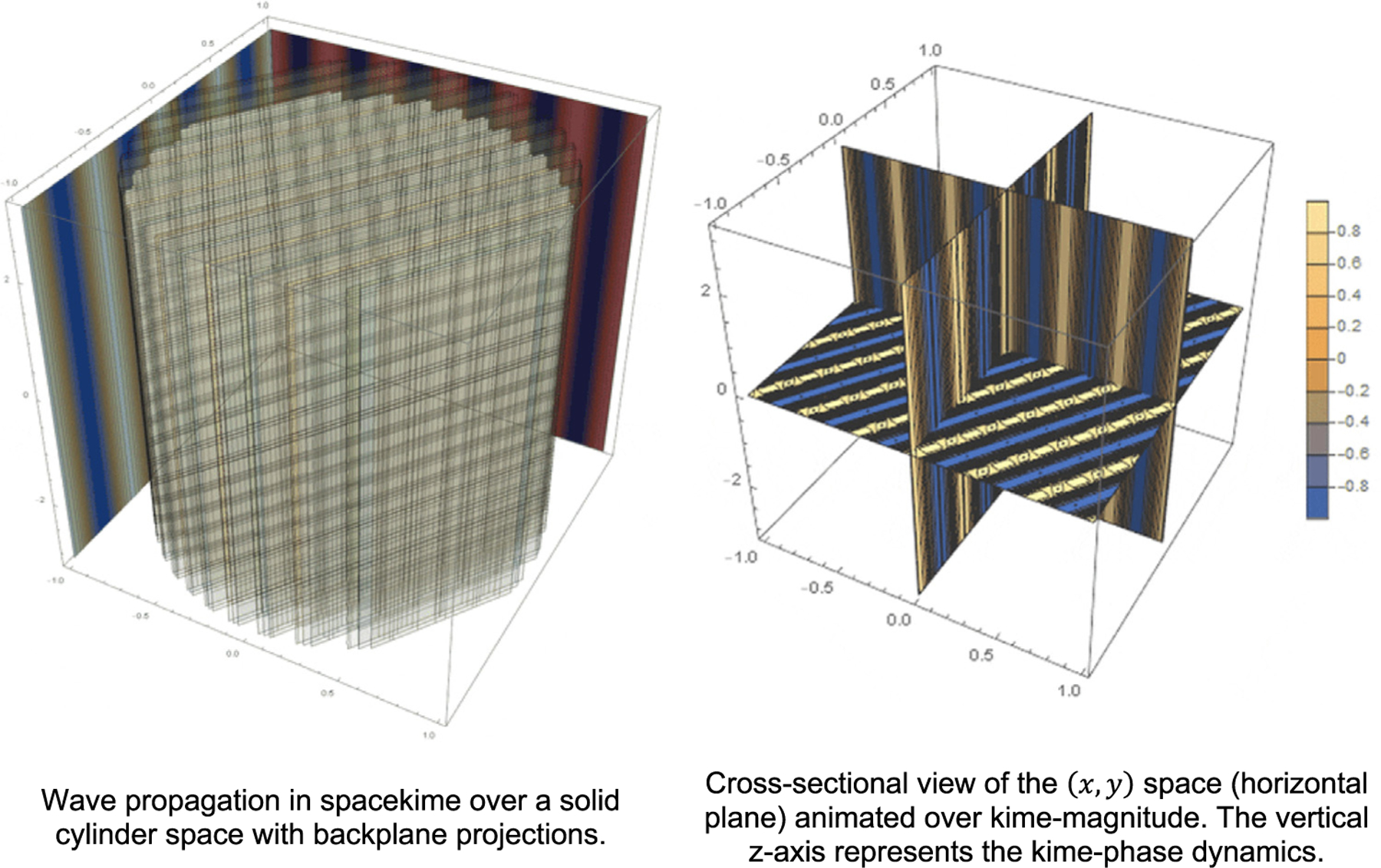
Example of the existence of a locally stable solution to the ultrahyperbolic wave equation in spacekime. The left and right figures illustrate alternative views and foliations of the 2D kime dynamics of the 5D spacekime wave projected onto a flat 2D (*x,* y) plane.

One specific solution illustrated on Fig. 1 is given by

(3.17) $$u(x,\kappa )=u\left(x_{1},x_{2},x_{3},t,\theta \right)=e^{2\pi ti(-2\cos\theta +3\sin\theta )}\times e^{2\pi i\left(-3x_{1}+2x_{2}\right)},$$

where $\eta =\left(\eta_{1},\eta_{2}\right)=(-2,3)$ and $\text{ξ}=\left(\xi_{1},\xi_{2},\xi_{3}\right)=(-3,2,0)$,$|\text{ξ}{|}^{2}=|\eta {|}^{2}=13$.

## 4. Polar coordinate and bounded spatial domain problem

Next, let us consider the solution to the ultrahyperbolic wave equation with an *initial condition*:

(4.1) $$\begin{array}{l} \Delta_{\kappa }u=\Delta_{x}u\\ {\left.u\right|}_{|\kappa |=1}(\kappa ,x)=f(\kappa ,x), \end{array}$$

where $f\in L^{2}$ and $\kappa \in {\mathbb{R}}^{2}$, $\left|\kappa \right|\leq 1$, $x\in {\left[-1,1\right)}^{d_{s}}$, under periodic spatial boundary conditions.

Since the initial condition is given at $\left|\kappa \right|=1$, it is natural to transform the equation into polar coordinates $\kappa_{1}=t\cos\phi$, $\kappa_{2}=t\sin\phi$,$\phi \in \left[-\pi ,\pi \right)$. The transformed equation (with slightly abusing the notation for the generic functions *u* and *f*) reads:

(4.2) $$\begin{array}{l} \partial_{t}^{2}u+\frac{1}{t}\partial_{t}u+\frac{1}{t^{2}}\partial_{\phi }^{2}u=\Delta_{x}\text{u},\\ u(1,\phi ,x)=f(\phi ,x). \end{array}$$

Note that *u* has to be periodic in *ϕ* and ***x***, so *f* is also periodic in *ϕ* and ***x***. Therefore, we may expand the Fourier series of *f*:

(4.3) $$f(\phi ,x)=\sum_{\eta \in \mathbb{Z},\xi \in {\mathbb{Z}}^{2}}C_{\eta ,\xi }\cdot e^{i\eta \phi }\cdot e^{2\pi i\langle \text{ξ},x\rangle }.$$

Since the differential equation $\partial_{t}^{2}u+\frac{1}{t}\partial_{t}u+\frac{1}{t^{2}}\partial_{\phi }^{2}u=\Delta_{x}u$ is linear, it suffices to solve each boundary value problem of the following form:

(4.4) $$\begin{array}{l} \partial_{t}^{2}v+\frac{1}{t}\partial_{t}v+\frac{1}{t^{2}}\partial_{\phi }^{2}v=\Delta_{x}v,\\ v(1,\phi ,x)=e^{i\eta \phi }\cdot e^{2\pi i\langle \text{ξ},x\rangle }, \end{array}$$

where η∈ℤ, $\text{ξ}\in {\mathbb{Z}}^{2}$ are arbitrary parameters.

As we indicated earlier, to solve these boundary value problems, we will consider separable solutions v(t,ϕ,x)=F(t,ϕ)⋅G(x). The differential equation $\partial_{t}^{2}v+\frac{1}{t}\partial_{t}v+\frac{1}{t^{2}}\partial_{\phi }^{2}v=\Delta_{x}v$ becomes:

(4.5) $$\left(\partial_{t}^{2}F+\frac{1}{t}\partial_{t}F+\frac{1}{t^{2}}\partial_{\phi }^{2}F\right)\cdot G=F\cdot \Delta_{x}G.$$

Therefore,

(4.6) $$\frac{\partial_{t}^{2}F+\frac{1}{t}\partial_{t}F+\frac{1}{t^{2}}\partial_{\phi }^{2}F}{F}=\frac{\Delta_{x}G}{G}=const.$$

Incorporating the boundary condition, we obtain

(4.7) $$F(1,\phi )\cdot G(x)=v(1,\phi ,x)=e^{i\eta \phi }\cdot e^{2\pi i\langle \text{ξ},x\rangle }.$$

Hence, we have

(4.8) $$G(x)=e^{2\pi i\langle \text{ξ},x\rangle },F(1,\phi )=e^{i\eta \phi },$$

and

(4.9) $$\frac{\partial_{t}^{2}F+\frac{1}{t}\partial_{t}F+\frac{1}{t^{2}}\partial_{\phi }^{2}F}{F}=\frac{\Delta_{x}G}{G}=-4\pi^{2}|\text{ξ}{|}^{2}.$$

Let us try to solve for *F* this PDE

(4.10) $$\begin{array}{l} \partial_{t}^{2}F+\frac{1}{t}\partial_{t}F+\frac{1}{t^{2}}\partial_{\phi }^{2}F+4\pi^{2}|\text{ξ}{|}^{2}F=0,\\ F(1,\phi )=e^{i\eta \phi }, \end{array}$$

using the standard method of solving Helmholtz equations.^19,20^ Consider $F(t,\phi )=\sum_{j}F_{j}(t,\phi )$, where each component can be separated into $F_{j}(t,\phi )=T_{j}(t)\cdot A_{j}(\phi )$ as specified below. Then, the equation for each *F*_*j*_ becomes:

(4.11) $$T_{j}^{\prime \prime }A_{j}+\frac{1}{t}T_{j}^{\prime }A_{j}+\frac{1}{t^{2}}T_{j}A_{j}^{\prime \prime }+4\pi^{2}|\xi {|}^{2}T_{j}A_{j}=0.$$

Dividing by *T*_*j*_*A*_*j*_, we have

(4.12) $$\frac{T_{j}^{\prime \prime }}{T_{j}}+\frac{1}{t}\frac{T_{j}^{\prime }}{T_{j}}+\frac{1}{t^{2}}\frac{A_{j}^{\prime \prime }}{A_{j}}+4\pi^{2}|\xi {|}^{2}=0.$$

Multiplying by *t*^2^, we obtain

(4.13) $$t^{2}\frac{T_{j}^{\prime \prime }}{T_{j}}+t\frac{T_{j}^{\prime }}{T_{j}}+4\pi^{2}|\xi {|}^{2}t^{2}=-\frac{A_{j}^{\prime \prime }}{A_{j}}=const.$$

Since *A*_*j*_ (*ϕ*) is periodic, and *F* is decomposed by frequencies, we may take *A*_*j*_ (*ϕ*) = *e*^*ijϕ*^. Then,

(4.14) $$t^{2}\frac{T_{j}^{\prime \prime }}{T_{j}}+t\frac{T_{j}^{\prime }}{T_{j}}+4\pi^{2}|\xi {|}^{2}t^{2}=-\frac{A_{j}^{\prime \prime }}{A_{j}}=j^{2}$$

and hence

(4.15) $$t^{2}\frac{T_{j}^{\prime \prime }}{T_{j}}+t\frac{T_{j}^{\prime }}{T_{j}}+4\pi^{2}|\xi {|}^{2}t^{2}=j^{2}.$$

Dividing by *t*^2^ and multiplying by *T*_*j*_, we obtain the Bessel equation:

(4.16) $$T_{j}^{\prime \prime }+\frac{1}{t}T_{j}^{\prime }+\left(4\pi^{2}|\xi {|}^{2}-\frac{j^{2}}{t^{2}}\right)T_{j}=0.$$

The order *j* Bessel function of the first kind, *J*_*j*_, solves the above equation and does not have singularity at *t* = 0,$T_{j}(t)=J_{j}(2\pi |\xi |t)$. Substituting the boundary condition that *T*_*j*_ (1) = 1, we have

(4.17) $$F(t,\phi )=\sum_{j}T_{j}(t)A_{j}(\phi )=\sum_{j=-\infty }^{\infty }\frac{J_{j}(2\pi |\xi |t)}{J_{j}(2\pi |\xi |)}\cdot e^{ij\phi }.$$

Comparing with the boundary condition $F(1,\phi )=e^{i\eta \phi }$ yields that only the j=η frequency remains in the definition

(4.18) $$F(t,\phi )=\frac{J_{\eta }(2\pi |\xi |t)}{J_{\eta }(2\pi |\xi |)}\cdot e^{i\eta \phi }.$$

Finally, combining with *G*(***x***), we have that the solution to the original boundary value problem

(4.19) $$\partial_{t}^{2}u+\frac{1}{t}\partial_{t}u+\frac{1}{t^{2}}\partial_{\phi }^{2}u=\Delta_{x}u\;u(1,\phi ,x)=f(\phi ,x)=\sum_{\eta \in \mathbb{Z},\text{ξ}\in {\mathbb{Z}}^{2}}C_{\eta ,\xi }\cdot e^{i\eta \phi }\cdot e^{2\pi i\langle \text{ξ},x\rangle }$$

may be expressed as a linear combination of different orders Bessel functions of the first kind

(4.20) $$u(t,\phi ,x)=\sum_{\eta \in \mathbb{Z},\xi \in {\mathbb{Z}}^{2}}C_{\eta ,\xi }\cdot e^{i\eta \phi }\cdot \frac{J_{\eta }(2\pi |\xi |t)}{J_{\eta }(2\pi |\xi |)}\cdot e^{2\pi i\langle \text{ξ},x\rangle }.$$

**Example.** Assume the boundary condition is given in terms of the first order Bessel function of the first kind,

(4.21) $$u(1,\phi ,x)=e^{i\phi }\cdot J_{1}(10\pi )\cdot e^{6\pi ix_{1}-8\pi ix_{2}}.$$

Then, the solution to the boundary value problem may be expressed as

(4.22) $$u(t,\phi ,x)=e^{i\phi }\cdot J_{1}(10\pi t)\cdot e^{6\pi ix_{1}-8\pi ix_{2}}.$$

## 5. Spherical coordinate and bounded spatial domain problem

Next, we will consider the problem

(5.1) $$\Delta_{\kappa }u=\Delta_{x}u,$$

where again $f\in L^{2}$ and $\kappa \in {\mathbb{R}}^{3}$, $\left|\kappa \right|\leq 1$, $x\in {\left[-\pi ,\pi \right)}^{d_{s}}$, periodic spatial boundary conditions.

Since the boundary condition is of radial form, i.e., $\left|\kappa \right|=1$, we can apply a transformation to spherical coordinates

(5.2) $$\kappa_{1}=t\sin\theta \cos\varphi ,\kappa_{2}=t\sin\theta \sin\varphi ,\kappa_{3}=t\cos\theta ,\;\theta \in [0,\pi ],\;\varphi \in [0,2\pi ].$$

The Laplacian can be transformed into

(5.3) $$\Delta_{\kappa }=\frac{1}{t^{2}}\frac{\partial }{\partial t}\left(t^{2}\frac{\partial }{\partial t}\right)+\frac{1}{t^{2}\sin^{2}\theta }\frac{\partial^{2}}{\partial \varphi^{2}}+\frac{1}{t^{2}\sin\theta }\frac{\partial }{\partial \theta }\left(\sin\theta \frac{\partial }{\partial \theta }\right).$$

As we require that *f* is periodic in ***x***, we may take the discrete Fourier series expansion to obtain

(5.4) $${\left.f(\kappa ,x)\right|}_{|\kappa |=1}=\sum_{\text{ξ}\in {\mathbb{Z}}^{2}}C_{\xi }\cdot e^{i\langle \text{ξ},x\rangle }\cdot K_{\text{ξ}}(\theta ,\varphi )$$

By linearity, it suffices to solve the problem

(5.5) $$\begin{array}{l} \Delta_{\kappa }u=\Delta_{x}u\\ {\left.u\right|}_{|\kappa |=1}=e^{i\langle \text{ξ},x\rangle }\cdot K_{\text{ξ}}(\theta ,\varphi ). \end{array}$$

We will restrict our solution space to separable solutions. Specifically, we will consider

(5.6) $$u(t,\theta ,\phi ,x)=\underset{F(t,\theta ,\varphi )}{\underset{︸}{R(t)\cdot \underset{\Phi (\varphi )\cdot \Theta (\theta )}{\underset{︸}{Y(\varphi ,\theta )}}}}\cdot G(x).$$

Applying the separable solution ansatz, we obtain

(5.7) $$\frac{\Delta_{\kappa }F}{F}=\frac{\Delta_{x}G}{G}=const.$$

The boundary condition $u(1,\theta ,\phi ,x)=e^{i\langle \text{ξ},x\rangle }\cdot K_{\text{ξ}}(\theta ,\varphi )$ means that $G(x)=e^{i\langle \text{ξ},x\rangle }$. Thus,

(5.8) $$\frac{\Delta F}{F}=\frac{\Delta_{x}G}{G}=-|\text{ξ}{|}^{2}.$$

This yields the Helmholtz’s equation for *F* as a specialization for the general Sturm–Liouville problem

(5.9) $$\begin{array}{l} \Delta_{\kappa }F=-|\text{ξ}{|}^{2}\cdot F\\ F(1,\theta ,\varphi )=K_{\text{ξ}}(\theta ,\varphi ). \end{array}$$

Different choices of $\text{ξ}\in {\mathbb{Z}}^{2}$ correspond to different eigenfunctions *F*_ξ_, and since the boundary value problem is self-adjoint,^1^ we may write the *general solution* as a linear combination of orthonormal eigenfunctions in the *L*^2^ space:

(5.10) $$u(t,\theta ,\varphi ,x)=\sum_{\text{ξ}\in {\mathbb{Z}}^{2}}C_{\xi }\cdot e^{i\langle \text{ξ},x\rangle }F_{\text{ξ}}(t,\theta ,\varphi ).$$

The solution has to respect the boundary problem. Hence,

(5.11) $$u(1,\theta ,\varphi ,x)={\left.f(\kappa ,x)\right|}_{|\kappa |=1}=\sum_{\text{ξ}\in {\mathbb{Z}}^{2}}C_{\xi }\cdot e^{i\langle \text{ξ},x\rangle }F_{\text{ξ}}(1,\theta ,\varphi ),$$

where

(5.12) $$\begin{array}{l} C_{\xi }=\frac{{\langle e^{i\langle \text{ξ},x\rangle }F_{\text{ξ}}(1,\theta ,\varphi ),{\left.f(\kappa ,x)\right|}_{|\kappa |=1}\rangle }_{L^{2}}}{{\langle e^{i\langle \text{ξ},x\rangle }F_{\text{ξ}}(1,\theta ,\varphi ),e^{i\langle \text{ξ},x\rangle }F_{\text{ξ}}(1,\theta ,\varphi )\rangle }_{L^{2}}}\\ \;=\frac{{\langle e^{i\langle \text{ξ},x\rangle }F_{\text{ξ}}(1,\theta ,\varphi ),{\left.f(\kappa ,x)\right|}_{|\kappa |=1}\rangle }_{L^{2}}}{{\left\|e^{i\langle \text{ξ},x\rangle }F_{\text{ξ}}(1,\theta ,\varphi )\right\|}_{L^{2}}}. \end{array}$$

In spherical coordinates, we can formally solve the eigenvalue problem within a ball under the following assumptions:

(5.13) $$\begin{array}{l} \Delta_{\kappa }F=-|\xi {|}^{2}\cdot F,\\ R(t)\cdot \underset{Y(\varphi ,\theta )}{\underset{︸}{\Phi (\varphi )\cdot \text{Θ}(\theta )}}=F(t,\theta ,\varphi ),\\ F(1,\theta ,\phi )=K_{\text{ξ}}(\theta ,\varphi ). \end{array}$$

Let us first separate out the radial part of the solution:

(5.14) $$\begin{array}{l} \frac{1}{t^{2}}\frac{\partial }{\partial t}\left(t^{2}\frac{\partial }{\partial t}(RY)\right)+\frac{1}{t^{2}\sin^{2}\theta }\frac{\partial^{2}}{\partial \varphi^{2}}(RY)+\frac{1}{t^{2}\sin\theta }\frac{\partial }{\partial \theta }\left(\sin\theta \frac{\partial }{\partial \theta }(RY)\right)\\ =-|\text{ξ}{|}^{2}RY,\\ \underset{-|\xi {|}^{2}RY+\mu RY\frac{1}{t^{2}}}{\underset{︸}{\frac{Y}{t^{2}}\frac{\partial }{\partial t}\left(t^{2}\frac{\partial }{\partial t}(R)\right)}}+\underset{-\mu RY\frac{1}{t^{2}}}{\underset{︸}{R\left(\frac{1}{t^{2}\sin^{2}\theta }\frac{\partial^{2}}{\partial \varphi^{2}}(Y)+\frac{1}{t^{2}\sin\theta }\frac{\partial }{\partial \theta }\left(\sin\theta \frac{\partial }{\partial \theta }(Y)\right)\right)}}\\ =-|\text{ξ}{|}^{2}RY, \end{array}$$

(5.15) $$\left|\begin{array}{l} \frac{1}{t^{2}}\frac{\partial }{\partial t}\left(t^{2}\frac{\partial }{\partial t}(R)\right)=(\overset{\text{eigenvalue }}{\overset{︷}{\frac{\mu }{t^{2}}-|\xi {|}^{2}}})R\\ \frac{1}{\sin^{2}\theta }\frac{\partial^{2}}{\partial \varphi^{2}}(Y)+\frac{1}{\sin\theta }\frac{\partial }{\partial \theta }\sin\theta \frac{\partial }{\partial \theta }(Y)=-\mu Y \end{array}\right.$$

We further separate out the second equation Y(φ,θ)=Φ(φ)⋅Θ(θ):

(5.16) $$\frac{1}{\sin^{2}\theta }\frac{\partial^{2}}{\partial \varphi^{2}}(\Phi \Theta )+\frac{1}{\sin\theta }\frac{\partial }{\partial \theta }\left(\sin\theta \frac{\partial }{\partial \theta }(\Phi \Theta )\right)=-\mu \Phi \Theta \;\underset{-v\frac{\Theta }{\sin^{2}\theta }\Phi }{\underset{︸}{\frac{\Theta }{\sin^{2}\theta }\frac{\partial^{2}}{\partial \varphi^{2}}(\Phi )}}+\underset{-\mu \Phi \theta +v\frac{\theta }{\sin^{2}\theta }\Phi }{\underset{︸}{\frac{\Phi }{\sin\theta }\frac{\partial }{\partial \theta }\left(\sin\theta \frac{\partial }{\partial \theta }(\Theta )\right)}}=-\mu \Phi \Theta ,$$

(5.17) $$\left|\begin{array}{c} \Phi^{\prime \prime }=\overset{\text{eigenvalue }}{\overset{︷}{-v}}\Phi \\ \sin\theta \frac{\partial }{\partial \theta }\left(\sin\theta \frac{\partial }{\partial \theta }(\Theta )\right)=-\mu \Theta \sin^{2}\theta +\nu \Theta =\underset{\text{eigenvalue}}{\underset{︸}{\left(-\mu \;\sin^{2}\theta +v\right)}}\Theta . \end{array}\right.$$

We now shall solve the three eigenvalue problems in Eqs. (5.15) and (5.17) with one independent variable. The reasoning above only requires us to find an orthonormal eigenfunction basis *F*_ξ_. The solution to the first equation in (5.17) is the easiest to obtain,

(5.18) $$\Phi =A_{v}\cos(v\varphi )+B_{v}\sin(v\varphi )=A_{m}\cos(m\varphi )+B_{m}\sin(m\varphi ).$$

Next, we turn to the second equation in (5.17)

(5.19) $$\sin\theta \frac{\partial }{\partial \theta }\left(\sin\theta \frac{\partial }{\partial \theta }(\Theta )\right)=\underset{\text{eigenvalue }}{\underset{︸}{\left(-\mu \sin^{2}\theta +v\right)}}\Theta ,\;\theta \in (0,\pi ).$$

Applying the coordinate transformation $t=\cos\theta \Rightarrow dt=-\sin\theta d\theta \Rightarrow \frac{d}{d\theta }=-\sin\theta \frac{d}{dt}$,

(5.20) $$\begin{array}{l} \theta \in (0,\pi )\Rightarrow \sin\theta \in (0,1]\Rightarrow \sin\theta =\sqrt{1-t^{2}},\\ \sin\theta \frac{\partial }{\partial \theta }\left(\sin\theta \frac{\partial }{\partial \theta }(\Theta )\right)=\sin\theta \frac{d}{d\theta }\left(\sin\theta \frac{d}{d\theta }(\Theta )\right)\\ \;=-\sin^{2}\theta \frac{d}{dt}\left(\sin\theta \left(-\sin\theta \frac{d}{dt}(\Theta )\right)\right)=\\ \;-\left(1-t^{2}\right)\frac{d}{dt}\left(-\left(1-t^{2}\right)\Theta^{\prime }\right)={\left(1-t^{2}\right)}^{2}\Theta^{\prime \prime }-\left(1-t^{2}\right)2t\Theta^{\prime }. \end{array}$$

This differential equation resolves to:

(5.21) $$\begin{array}{l} {\left(1-t^{2}\right)}^{2}\Theta^{\prime \prime }-\left(1-t^{2}\right)2t\Theta^{\prime }=-\mu \left(1-t^{2}\right)\Theta +v\text{Θ,}\\ t\in (-1,1)\Rightarrow \left(1-t^{2}\right)\neq 0. \end{array}$$

Thus,

(5.22) $$\left(1-t^{2}\right)\Theta^{\prime \prime }-2t\Theta^{\prime }+\left(\mu -\frac{v}{1-t^{2}}\right)\Theta =0.$$

The solution is the generalized Legendre polynomial with parameters *μ* = *n*(*n*+1) and *v* = *m*:

(5.23) $$\Theta (\theta )=P_{n}^{m}(\cos\theta ),$$

where the integer indices $\left|m\right|\leq n$ and $P_{n}^{m}$ are the associated Legendre polynomials are:

(5.24) $$P_{n}^{m}(x)=\frac{{\left(-1\right)}^{m}}{2^{n}n!}{\left(1-x^{2}\right)}^{m/2}\frac{d^{n+m}}{dx^{n+m}}{\left(x^{2}-1\right)}^{n}.$$

**Remark:** The functions $Y_{m,n}(\varphi ,\theta )=\Theta (\theta )\Phi (\varphi )=e^{im\varphi }P_{n}^{m}(\cos\theta )$, n∈ℕ, m=−n,−n+1,…,n, are called spherical harmonics of order *n*.

It remains to solve the radial part Eq. (5.15) with *μ* = *n*(*n*+1):

(5.25) $$\begin{array}{l} \frac{1}{t^{2}}\frac{\partial }{\partial t}\left(t^{2}\frac{\partial }{\partial t}(R)\right)-\overset{\text{eigenvalue }}{\overset{︷}{\left(\frac{\mu }{t^{2}}-|\xi {|}^{2}\right)}}R\\ \;=\frac{1}{t^{2}}\frac{d}{dt}\left(t^{2}R^{\prime }\right)-\left(\frac{n(n+1)}{t^{2}}-|\xi {|}^{2}\right)R=\\ \;R^{\prime \prime }+\frac{2}{t}R^{\prime }-\left(\frac{n(n+1)}{t^{2}}-|\xi {|}^{2}\right)R=0. \end{array}$$

We substitute $\rho =\sqrt{t}R\Rightarrow R=\frac{1}{\sqrt{t}}\rho \Rightarrow R^{\prime }=\frac{-1}{2t^{\frac{3}{2}}}\rho +\frac{1}{\sqrt{t}}\rho^{\prime }\Rightarrow R^{\prime \prime }=\frac{3}{4t\frac{5}{2}}\rho +\frac{-1}{t^{\frac{3}{2}}}\rho^{\prime }+\frac{1}{\sqrt{t}}\rho^{\prime \prime }$:

(5.26) $$\begin{array}{l} \frac{3}{4t^{\frac{5}{2}}}\rho +\frac{-1}{t^{\frac{3}{2}}}\rho^{\prime }+\frac{1}{\sqrt{t}}\rho^{\prime \prime }+\frac{-1}{t^{\frac{5}{2}}}\rho +\frac{2}{t^{\frac{3}{2}}}\rho^{\prime }-\left(\frac{n(n+1)}{t^{2}}-|\xi {|}^{2}\right)\frac{1}{\sqrt{t}}\rho =0\\ \;\;\;\;\Rightarrow t^{2}\rho^{\prime \prime }+t\rho^{\prime }+\left(t^{2}|\xi {|}^{2}-{\left(n+\frac{1}{2}\right)}^{2}\right)\rho =0. \end{array}$$

Using S(s)=ρ(t) and $s=t|\xi |\;\Rightarrow \;|\xi |\frac{d}{ds}=\frac{d}{dt}\Rightarrow \;|\xi |S^{\prime }=\rho^{\prime }\Rightarrow \;|\xi {|}^{2}S^{\prime \prime }=\rho^{\prime \prime }$. Therefore,

(5.27) $$s^{2}S^{\prime \prime }+sS^{\prime }+\left(s^{2}-{\left(n+\frac{1}{2}\right)}^{2}\right)S=0.$$

The solution to this Bessel differential equation is the Bessel function of order $n+\frac{1}{2}$:

(5.28) $$\begin{array}{l} R(t)=\frac{1}{\sqrt{t}}\rho (t)=\frac{1}{\sqrt{t}}S\left(\underset{s}{\underset{︸}{t|\xi |}}\right)=\frac{1}{\sqrt{t}}J_{\left(n+\frac{1}{2}\right)}(|\xi |t)\\ \;\;+\underset{\text{As }R(t)\text{ is bounded at }t=0}{\underset{︸}{\frac{1}{\sqrt{t}}J_{-\left(n+\frac{1}{2}\right)}(|\xi |t)}}. \end{array}$$

Thus,

(5.29) $$\begin{array}{l} F_{\xi }(t,\theta ,\varphi )=R(t)\cdot \Phi (\varphi )\cdot \Theta (\theta )=\frac{1}{\sqrt{t}}J_{\left(n+\frac{1}{2}\right)}(|\xi |t)P_{n}^{m}(\cos\theta )\left(A_{m}\cos(m\varphi )\right.\\ \left.\;+B_{m}\sin(m\varphi )\right),\;n=0,1,2,3,\ldots \;\text{and}\;m=0,1,2,3,\ldots ,n, \end{array}$$

(5.30) $$\begin{array}{l} u(t,\theta ,\varphi ,x)=\sum_{n=0}^{\infty }\sum_{m=0}^{n}\sum_{\xi \in {\mathbb{Z}}^{2}}C_{\xi ,n,m}\cdot e^{i\langle \text{ξ},x\rangle }\frac{1}{\sqrt{t}}J_{\left(n+\frac{1}{2}\right)}\\ \;\;\;\;\;\times (|\text{ξ}|t)P_{n}^{m}(\cos(\theta ))\left(A_{m}\cos(m\varphi )+B_{m}\sin(m\varphi )\right)=\\ \;\;\;\;\;\sum_{n=0}^{\infty }\sum_{m=0}^{n}\sum_{\xi \in {\mathbb{Z}}^{2}}C_{\xi ,n,m}\cdot e^{i\langle \text{ξ},x\rangle }\frac{1}{\sqrt{t}}J_{\left(n+\frac{1}{2}\right)}(\left|\text{ξ}\right|t)P_{n}^{m}(\cos(\theta ))e^{im\varphi }. \end{array}$$

Let us ensure that the boundary conditions hold. The scalar factors should satisfy:

(5.31) $$\begin{array}{l} C_{\xi ,n,m}=\frac{{\langle e^{i\langle \text{ξ},x\rangle }F_{\text{ξ}}(1,\theta ,\varphi ),{\left.f(\kappa ,x)\right|}_{|\kappa |=1}\rangle }_{L^{2}}}{{\langle e^{i\langle \text{ξ},x\rangle }F_{\text{ξ}}(1,\theta ,\varphi ),e^{i\langle \text{ξ},x\rangle }F_{\text{ξ}}(1,\theta ,\varphi )\rangle }_{L^{2}}}\\ \;\;=\frac{{\langle e^{i\langle \text{ξ},x\rangle }F_{\text{ξ}}(1,\theta ,\varphi ),{\left.f(\kappa ,x)\right|}_{|\kappa |=1}\rangle }_{L^{2}}}{{\left\|e^{i\langle \text{ξ},x\rangle }F_{\text{ξ}}(1,\theta ,\varphi )\right\|}_{L^{2}}^{2}}\\ \;\;F_{\text{ξ}}(1,\theta ,\varphi )=R(1)\cdot \Phi (\varphi )\cdot \Theta (\theta )=J_{\left(n+\frac{1}{2}\right)}(|\text{ξ}|)P_{n}^{m}(\cos\theta )e^{im\varphi }. \end{array}$$

Let us illustrate this solution formula with a couple of simple examples, the detailed derivation is presented in Appendix A, Table 2.

When the boundary condition is independent of *φ*, the ultrahyperbolic wave equation with (3D) spherical time coordinates has a solution that is also independent of *φ*. Note that in the previous example, the solution *u* is independent of *φ*. This is because the boundary condition $u\left|{}_{\left|\kappa \right|=1}\right.$ is independent of *φ*, and it follows from the rotational invariance of the Laplace operator that the solution has to be independent of *φ* as well. The details are provided in the following proposition.

**Proposition 1.** *When the boundary condition is independent of φ, then the solution will also be independent of φ.*

**Proof of proposition.** Assume the boundary condition depends only on ***x*** and *θ*. Let ***Ф*** be a rotation in the *φ* direction (and hence an orthogonal transformation). Then, the boundary condition is invariant under ***Ф***, and so the solution is also independent of *φ*.

**Lemma 2.** *Let $D\subset {\mathbb{R}}^{n}$ be an open set, f* ∈ *C*^2^ (*D*)*, and let* ***Ф*** ∈ *O* (*n*) *be an orthogonal (linear) transformation leaving D invariant (i.e.,* ***Ф*** (*D*) = *D). Then,*

(5.32) Δ(f∘Φ)=(Δf)∘Φ.

**Proof.** Let *u*_1_ be a solution to the ultrahyperbolic wave equation with ${\left.u_{1}\right|}_{|\kappa |=1}=f(x,\theta )$. Let ***Ф*** be a rotation in the direction *ϕ* and $u_{2}=u_{1}\circ \Phi$.

Then,${\left.u_{2}\right|}_{|\kappa |=1}=\Phi f(x,\theta )=f(x,\theta )={\left.u_{1}\right|}_{|\kappa |=1}$. Note that

(5.33) $$\Delta_{\kappa }u_{2}=\Delta_{\kappa }u_{1}=\Delta_{x}u_{1}=\Delta_{x}u_{2}.$$

Therefore, *u*_2_ also solves the equation and so *u*_2_ = *u*_1_.

## 6. Cartesian coordinate and unbounded spatial domain problem

For the non-periodic Cauchy value problem, non-integer (real and complex) spatial and temporal frequency vectors are possible, e.g.,

(6.1) $$\begin{array}{l} \eta =\left(\eta_{1},\eta_{2}\right)=(\pi \sqrt{2},\pi \sqrt{2})\;and\\ \;\text{ξ}=\left(\xi_{1},\xi_{2},\xi_{3}\right)=(\pi ,\pi \sqrt{3},0),\;where\;|\text{ξ}{|}^{2}=|\eta {|}^{2}=4\pi^{2}. \end{array}$$

Next, we examine the existence, stability, determinism, and uniqueness of local and global solutions to the wave equation Cauchy initial value problem.^21,22^ For non-local constraints, the Cauchy initial value problem on co-dimension 1 hypersurfaces is well-posed and has global unique solutions in Sobolev spaces *H*^*m*^.

However, the Cauchy initial value problem, formulated on higher co-dimension hypersurfaces in terms of a finite number of derivatives of the data, is globally ill-posed. It does not permit global solutions, but allows for locally unique solutions in neighborhoods of the initial hypersurfaces.

In essence, the general lack of global stability and uniqueness for the ultrahyperbolic wave equation, with Cauchy initial value formulation, can be resolved by imposing non-local constraints naturally arising from the field equations. Such non-local constraints may preserve stability of the solutions but not their determinism or uniqueness.

The Cauchy initial value problem associated with the ultrahyperbolic wave equation can be formulated as a linear constraint representing a hypersurface of co-dimension 1. Co-dimension 1 refers to the time variable (e.g.,$\kappa =\left(\kappa_{1},\kappa_{2}\right)=te^{i\theta }$) split of the time domain in two subspaces ${\mathbb{R}}^{d_{t}}⊇D_{t}=D_{1}\overset{\cdot }{\cup }D_{1}^{c}$. The one-dimensional *D*_1_ subspace represents κ_1_ – the dynamic evolution time (e.g., *t*). The complementary (*d*_*t*_ – 1) dimensional subspace $D_{1}^{c}$ consist of the remaining independent time-like dimensions (e.g., {κ_–1_} or *θ*), where

$$\kappa =\left(\kappa_{1},\underset{\kappa_{-1}}{\underset{︸}{\kappa_{2},\ldots ,\kappa_{d_{t}}}}\right)\in D_{t}.$$

Along the first time-like coordinate, *κ*_1_, the evolution of the ultrahyperbolic wave equation with Cauchy initial conditions (Cauchy data) is governed by:

(6.2) $$\begin{array}{l} \sum_{i=1}^{d_{s}}\partial_{x_{i}}^{2}u\equiv \Delta_{x}u=\Delta_{\kappa }u\equiv \sum_{i=1}^{d_{t}}\partial_{\kappa_{i}}^{2}u\\ \left|\begin{array}{l} u\left(\underset{x\in D_{s}}{\underset{︸}{x}},\underset{\kappa \in D_{t}}{\underset{︸}{0,\kappa_{-1}}}\right)=f\left(x,\kappa_{-1}\right)\\ \;\partial_{\kappa_{1}}u\left(x,0,\kappa_{-1}\right)=g\left(x,\kappa_{-1}\right). \end{array}\right. \end{array}$$

Typically, the initial constraints are formulated in terms of *κ*_1_, a.k.a. the direction of (temporal) evolution, as restrictions over the neighborhood $N=\left\{(x,\kappa )\in D_{s}\times D_{t}|\kappa_{1}=0\right\}$ representing a hypersurface subspace of dimension one less than that of the entire space, *D*_*s*_ × *D*_*t*_. Higher co-dimensional constraints are defined analogously using two or more time dimensions representing the temporal dynamics.

The Cauchy initial value problem depends on how much data (initial restrictions) are assumed or given a priori. For instance, one may fix the value of the potential function (zeroth derivative) and the first partial derivatives, or alternatively fix a finite number of partial derivatives of *u* (***x***, ***κ*)**, **o**n the neighborhood *N*, and require compatibility of the imposed constraints with the wave equation,$\Delta_{x}u=\Delta_{\kappa }u$.

The standard *Sobolev space* of functions is defined as the closure, *H*^*m*^, of the following function space:

(6.3) $$\begin{array}{l} H^{m}=\text{closure}\left\{f\left(x,\kappa_{-1}\right)\in C^{\infty }\left({\mathbb{R}}^{d_{s}}\times {\mathbb{R}}^{d_{t}-1}\right)\left|\|f{\|}_{m}^{2}\right.\right.\\ \left.\;\;\;\equiv \sum_{|\alpha |+|\beta |\leq m}\int {\left|\partial_{x}^{\alpha }\partial_{\kappa_{-1}}^{\beta }f\left(x,\kappa_{-1}\right)\right|}^{2}dxd\kappa_{-1}\right\}. \end{array}$$

As the spacekime equation can be expressed as $\partial_{\kappa_{1}}^{2}u=\Delta_{x}u-\Delta_{\kappa_{-1}}u$, we can define the *energy functional*, associated with potential function solution, as:

(6.4) $$E[u]\left(\kappa_{1}\right)=\frac{1}{2}\int \left[{\left|\partial_{\kappa_{1}}u\right|}^{2}+{\left|\nabla_{x}u\right|}^{2}-{\left|\nabla_{\kappa_{-1}}u\right|}^{2}\right]dxd\kappa_{-1}.$$

Recall that the column-vector (spatial) gradient is $\nabla_{x}u=\left(\frac{\partial u}{\partial_{x_{1}}},\frac{\partial u}{\partial_{x_{2}}},\right.{\left.\ldots ,\frac{\partial u}{\partial_{x_{d_{s}}}}\right)}^{\prime }$, where $\partial_{\kappa_{1}}(u)=\frac{\partial u}{\partial_{\kappa_{1}}}$. If $U(x,\kappa )=\nabla_{x}u-\nabla_{\kappa_{-1}}u\equiv \left(\nabla_{x}-\nabla_{\kappa_{-1}}\right)u$ and $V(x,\kappa )=\partial_{\kappa_{1}}u$, then integration by parts, in loose terms, yields $\int UV^{\prime }\;dxd\kappa =UV-\int U^{\prime }V\;dxd\kappa$.

Using this property along with the initial condition $\partial_{\kappa_{1}}u\left(x,0,\kappa_{-1}\right)=g\left(x,\kappa_{-1}\right)$, i.e., the partial derivative with respect to the dynamic’s parameter (*κ*_1_) of the periodic function is independent of the value of *κ*_1_, using Green’s identities, we conclude that:

(6.5) $$\begin{array}{l} \int \nabla_{x}u\cdot \partial_{\kappa_{1}}\nabla_{x}(u)d\kappa_{-1}dx\\ \;=\int \nabla_{x}u\cdot \nabla_{x}\left(\partial_{\kappa_{1}}u\right)d\kappa_{-1}dx\\ \;=\int_{D_{x}}\int_{D_{t_{-1}}}\nabla_{x}\left(\partial_{\kappa_{1}}u\right)\cdot \nabla_{x}ud\kappa_{-1}dx\\ \;=\int_{D_{t_{-1}}}\left(\underset{0,\text{u vanishes at infinity }}{\underset{︸}{\int_{\partial D_{x}}\partial_{\kappa_{1}}u\left(\nabla_{x}u\cdot \hat{\text{n}}\right)d\text{s}}}\right)d\kappa_{-1}\\ \;-\int_{D_{x}}\int_{D_{t_{-1}}}\left(\partial_{\kappa_{1}}u\right)\cdot \nabla_{x}^{2}ud\kappa_{-1}dx\\ \;=-\int \left(\partial_{\kappa_{1}}u\right)\cdot \Delta_{x}udxd\kappa_{-1}. \end{array}$$

Similarly,

(6.6) $$\int \nabla_{\kappa_{-1}}u\cdot \nabla_{\kappa_{-1}}\partial_{\kappa_{1}}(u)d\kappa_{-1}dx=-\int \left(\partial_{\kappa_{1}}u\right)\cdot \Delta_{\kappa_{-1}}udxd\kappa_{-1}.$$

When the dynamic evolution mapping

(6.7) $$\kappa_{1}\vec{\text{ℳ}}\left(\begin{array}{c} u\left(x,\kappa_{1},\kappa_{-1}\right)\\ \partial_{\kappa_{1}}u\left(x,\kappa_{1},\kappa_{-1}\right) \end{array}\right)$$

has a smooth first order derivative, $ℳ\in C^{1}\left({\mathbb{R}}_{\kappa_{1}}\to H^{1}\times H^{o}\right)$ then the energy functional is conserved along the solution path $u\left(\underset{x}{\underset{︸}{\;\;\;\cdot \;\;\;}},\kappa_{1},\underset{\kappa_{-1}}{\underset{︸}{\;\;\;\cdot \;\;\;}}\right)$.

That is Eq. (6.8) in Box I

Therefore, the energy is constant with respect to *κ*_1_

(6.9) $$\begin{array}{l} E[u]\left(\kappa_{1}\right)=E\left(u\left(\underset{x}{\underset{︸}{\cdot }},\kappa_{1},\underset{\kappa_{-1}}{\underset{︸}{\cdot }}\right)\right)\\ \;\;\;\;=E\left(u\left(\underset{x}{\underset{︸}{\cdot }},0,\underset{\kappa_{-1}}{\underset{︸}{\cdot }}\right)\right)=E[u](0). \end{array}$$

This validates that the energy is preserved along the (univariate, *κ*_1_) temporal evolution trajectory. However, the energy being preserved along *k*_1_ does not imply that the equation is well-posed. The reason is that the energy functional is not bounded below, and hence, we may have $\partial_{\kappa_{1}}u$, $\nabla_{K_{-1}}u$, $\nabla_{x}u$ be unbounded while the difference remains constant. Therefore, the energy functional does not control the *H*^1^-norm of the solution. In fact, the Cauchy initial value problem for the ultrahyperbolic wave equation is ill-posed in general.^21^

For this Cauchy initial value problem, the Fourier transform maps the spatio-temporal variables (***x*, *κ***_–1_), to their frequency counterparts (***ξ, η***_*–*1_) and we can examine the evolution operator.


(6.10) $$\left(\begin{array}{c} u_{o}\left(x,\kappa_{-1}\right)\\ u_{1}\left(x,\kappa_{-1}\right) \end{array}\right)=\left(\begin{array}{c} u\left(x,\kappa_{1},\kappa_{-1}\right)\\ \partial_{\kappa_{1}}u\left(x,\kappa_{1},\kappa_{-1}\right) \end{array}\right)\in H^{m+1}\times H^{m},$$

(6.11) $$\begin{array}{l} ℱ\left(\begin{array}{l} u_{o}\\ u_{1} \end{array}\right)=\hat{\left(\begin{array}{c} u_{o}\\ u_{1} \end{array}\right)}=\left(\begin{array}{c} {\hat{u}}_{o}\left(\text{ξ},\eta_{-1}\right)\\ {\hat{u}}_{1}\left(\text{ξ},\eta_{-1}\right) \end{array}\right)\\ \;\;\;=\int_{D_{x},D_{t_{-1}}}\left(\begin{array}{c} u_{o}\left(x,\kappa_{-1}\right)\\ u_{1}\left(x,\kappa_{-1}\right) \end{array}\right)e^{-2\pi i\langle \eta_{-1},\kappa_{-1}\rangle }\times e^{-2\pi i\langle \text{ξ},x\rangle }dxd\kappa_{-1}. \end{array}$$

We can thus derive solutions to the following Cauchy initial value problem

(6.12) $$\left(\begin{array}{l} u_{o}\\ u_{1} \end{array}\right)=\left(\begin{array}{l} u_{o}\left(x,\kappa_{-1}\right)\\ u_{1}\left(x,\kappa_{-1}\right) \end{array}\right)=\left(\begin{array}{l} u\left(x,\overset{\kappa_{1}}{\overset{︷}{0}},\kappa_{-1}\right)\\ \partial_{\kappa_{1}}u\left(x,\underset{\kappa_{1}}{\underset{︸}{0}},\kappa_{-1}\right) \end{array}\right)\in H^{m+1}\times H^{m}.$$

Case 1.$\left|\text{ξ}\right|\geq \left|\eta_{-1}\right|$, so $-|\text{ξ}{|}^{2}+{\left|\eta_{-1}\right|}^{2}\leq 0$. The general solutions to the ODE $\partial_{\kappa_{1}}^{2}\hat{u}=4\pi^{2}\left(-|\text{ξ}{|}^{2}+{\left|\eta_{-1}\right|}^{2}\right)$ with respect to *κ*_1_ is:

(6.13) $$\hat{u}=C_{1}\cdot \cos\left(2\pi \kappa_{1}\sqrt{|\text{ξ}{|}^{2}-{\left|\eta_{-1}\right|}^{2}}\right)+C_{2}\cdot \sin\left(2\pi \kappa_{1}\sqrt{|\text{ξ}{|}^{2}-{\left|\eta_{-1}\right|}^{2}}\right).$$

The solution to the initial value problem is therefore

(6.14) $$\begin{array}{l} \hat{u}\left(\text{ξ},\kappa_{1},\eta_{-1}\right)=\cos\left(2\pi \kappa_{1}\sqrt{|\text{ξ}{|}^{2}-{\left|\eta_{-1}\right|}^{2}}\right)\times {\hat{u}}_{o}\left(\text{ξ},\eta_{-1}\right)\\ \;\;\;\;\;+\frac{\sin\left(2\pi \kappa_{1}\sqrt{|\text{ξ}{|}^{2}-{\left|\eta_{-1}\right|}^{2}}\right)}{2\pi \sqrt{|\text{ξ}{|}^{2}-{\left|\eta_{-1}\right|}^{2}}}{\hat{u}}_{1}\left(\text{ξ},\eta_{-1}\right). \end{array}$$

Case 2.$\left|\text{ξ}\right|\geq \left|\eta_{-1}\right|$, so $-|\text{ξ}{|}^{2}+{\left|\eta_{-1}\right|}^{2}>0$. The general solutions to the ODE $\partial_{\kappa_{1}}^{2}\hat{u}=4\pi^{2}\left(-|\text{ξ}{|}^{2}+{\left|\eta_{-1}\right|}^{2}\right)$ with respect to *κ*_1_ is:

(6.15) $$\hat{u}=C_{1}\cdot \exp\left(2\pi \kappa_{1}\sqrt{|\eta_{-1}{|}^{2}-{\left|\text{ξ}\right|}^{2}}\right)+C_{2}\cdot \exp\left(-2\pi \kappa_{1}\sqrt{|\eta_{-1}{|}^{2}-{\left|\text{ξ}\right|}^{2}}\right).$$

The solution to the initial value problem is therefore

(6.16) $$\begin{array}{l} \hat{u}\left(\text{ξ},\kappa_{1},\eta_{-1}\right)=\cosh\left(2\pi \kappa_{1}\sqrt{|\eta_{-1}{|}^{2}-{\left|\text{ξ}\right|}^{2}}\right)\times {\hat{u}}_{o}\left(\text{ξ},\eta_{-1}\right)\\ \;\;\;\;\;+\frac{\sinh\left(2\pi \kappa_{1}\sqrt{|\eta_{-1}{|}^{2}-{\left|\text{ξ}\right|}^{2}}\right)}{2\pi \sqrt{|\eta_{-1}{|}^{2}-{\left|\text{ξ}\right|}^{2}}}{\hat{u}}_{1}\left(\text{ξ},\eta_{-1}\right). \end{array}$$

Then, the wave equation becomes $\partial_{\kappa_{1}}^{2}\hat{u}=\left(-|\text{ξ}{|}^{2}+{\left|\eta_{-1}\right|}^{2}\right)\hat{u}$, which has the following solutions:

(6.17) $$\left|\begin{array}{l} \begin{array}{c} \hat{u}\left(\text{ξ},\kappa_{1},\eta_{-1}\right)=\cos\left(\kappa_{1}\sqrt{|\text{ξ}{|}^{2}-{\left|\eta_{-1}\right|}^{2}}\right)\times {\hat{u}}_{o}\left(\text{ξ},\eta_{-1}\right)\\ +\frac{\sin\left(\kappa_{1}\sqrt{|\text{ξ}{|}^{2}-{\left|\eta_{-1}\right|}^{2}}\right)}{\sqrt{|\text{ξ}{|}^{2}-{\left|\eta_{-1}\right|}^{2}}}{\hat{u}}_{1}\left(\text{ξ},\eta_{-1}\right),|\text{ξ}|\geq \left|\eta_{-1}\right|\\ \hat{u}\left(\text{ξ},\kappa_{1},\eta_{-1}\right)=\cosh\left(\kappa_{1}\sqrt{{\left|\eta_{-1}\right|}^{2}-|\text{ξ}{|}^{2}}\right)\times {\hat{u}}_{o}\left(\text{ξ},\eta_{-1}\right)\\ +\frac{\sinh\left(\kappa_{1}\sqrt{{\left|\eta_{-1}\right|}^{2}-|\text{ξ}{|}^{2}}\right)}{\sqrt{{\left|\eta_{-1}\right|}^{2}-|\text{ξ}{|}^{2}}}{\hat{u}}_{1}\left(\text{ξ},\eta_{-1}\right),|\text{ξ}|<\left|\eta_{-1}\right|. \end{array} \end{array}\right.$$

The *Asgeirsson’s mean value theorem*^23,24^ states that the average of the potential function over the spatial domain is the same as its average over the temporal domain

(6.18) $$\int_{|x|=\rho }^{u}u(x,\kappa =0)dS(x)=\int_{|\kappa |=\rho }^{u}u(x=0,\kappa )dS(\kappa ),$$

where *p >* 0 is the radius of a Euclidean sphere in ${\mathbb{R}}^{d}$, $d\in \left\{d_{s},d_{t}\right\}$,*d****S*** is the surface area, and the potential function *u* represents a solution of the wave equation $\Delta_{x}u=\Delta_{\kappa }u$ over a neighborhood $N=\left\{\left.(x,\kappa )\in D_{s}\times D_{t}\subseteq {\mathbb{R}}^{d_{s}+d_{t}}\right|\left|x\right|+\left|\kappa \right|\leq \rho \right\}$.

The mean value theorem suggests why ensuring the existence and stability of solutions of the ultrahyperbolic wave equation requires some additional non-local constraints that may be derived by Fourier synthesis.^21,22^

The global stability of the solutions to the Cauchy data problem is guaranteed when |***ξ***| ≥ |***η***_*–*1_|, as the coefficients $\cos\left(2\pi \kappa_{1}\sqrt{|\text{ξ}{|}^{2}-{\left|\eta_{-1}\right|}^{2}}\right)$ and $\frac{\sin\left(2\pi \kappa_{1}\sqrt{|\text{ξ}{|}^{2}-{\left|\eta_{-1}\right|}^{2}}\right)}{2\pi \sqrt{|\text{ξ}{|}^{2}-{\left|\eta_{-1}\right|}^{2}}}$ are bounded for any *k*_1_. Thus, the solutions exist globally and are stable.

On the contrary, when |***ξ***| < |***η***_*–*1_|, we do not have global stability because the corresponding coefficients, sinh () and cosh (), are not bounded, and hence $\cosh\left(2\pi \kappa_{1}\sqrt{{\left|\eta_{-1}\right|}^{2}-|\text{ξ}{|}^{2}}\right)$ and $\sinh\left(2\pi \kappa_{1}\sqrt{{\left|\eta_{-1}\right|}^{2}-|\text{ξ}{|}^{2}}\right)$ will increase exponentially fast with *κ*_1_. Since the Fourier transform is 𝐿^2^ isometry, $\|u{\|}_{L^{2}}=\|\hat{u}{\|}_{L^{2}}$, and therefore, *𝑢* (***x, k***) is unbounded.

The connection between the Fourier-domain solution and spacetime solution can be expressed for each given $\hat{u}\left(\text{ξ},\kappa_{1},\eta_{-1}\right)\in L^{2}\left(d\text{ξ},d\eta_{-1}\right)$, by inverting the Fourier transform to obtain the corresponding spacetime solution:

(6.19) $$u\left(x,\kappa_{1},\kappa_{-1}\right)=\int_{D_{s},D_{t_{-1}}}\hat{u}\left(\text{ξ},\kappa_{1},\eta_{-1}\right)\cdot e^{2\pi i\langle x,\text{ξ}\rangle }\times e^{2\pi i\langle \kappa_{-1},\eta_{-1}\rangle }d\text{ξ}d\eta_{-1}.$$

Earlier, we noted that the energy, 𝐸, does not define a proper norm on the Sobolev space of solutions of the evolution equations. However, the Cauchy initial value evolution operator

(6.20) $$v=\left(\begin{array}{c} v_{o}\\ v_{1} \end{array}\right)\in \underset{\text{phase space }}{\underset{︸}{X\equiv H^{m+1}\times H^{m}}}$$

naturally leads to an energy norm defined by:

(6.21) $$\begin{array}{l} \|v{\|}_{X}^{2}\equiv \int_{|\text{ξ}|\geq \left|\eta_{-1}\right|}\left(|\text{ξ}{|}^{2}-{\left|\eta_{-1}\right|}^{2}\right){\left|{\hat{v}}_{o}\left(\text{ξ},\eta_{-1}\right)\right|}^{2}d\text{ξ}d\eta_{-1}\\ +\int_{|\text{ξ}|<\left|\eta_{-1}\right|}\left[{\left|\eta_{-1}\right|}^{2}-|\text{ξ}{|}^{2}\right]{\left|{\hat{v}}_{o}\left(\text{ξ},\eta_{-1}\right)\right|}^{2}d\text{ξ}d\eta_{-1}\\ +\int {\left|{\hat{v}}_{1}\left(\text{ξ},\eta_{-1}\right)\right|}^{2}d\text{ξ}d\eta_{-1}. \end{array}$$

This modified-energy, $\|v{\|}_{X}^{2}$, rectifies the problems with the original energy, and satisfies the scalability, sub-additivity, and point-separability conditions for all norms. Using this modified energy, we can split the phase space into:

(6.22) $$X\equiv H^{m+1}\times H^{m}=\left(X^{\sigma }\backslash X^{\tau }\right)\overset{\cdot }{\cup }\left(X^{\tau }\backslash X^{\sigma }\right)\overset{\cdot }{\cup }\left(X^{\tau }\cap X^{\sigma }\right).$$

The entire domain of the evolution in terms of the temporal dynamics in terms of $\kappa_{1}=t\in {\mathbb{R}}^{+}$, can be partitioned into three subspaces: *center stable* space, *X*^σ^, the *center unstable* space, *X*^τ^, and the *center* space, $X^{c}=X^{\tau }\cap X^{\sigma }$, which are defined by:

(6.23) $$\begin{array}{l} X^{\sigma }=\left\{v=\left(\begin{array}{c} v_{o}\\ v_{1} \end{array}\right)\in \left.X\right|\left({\hat{v}}_{o}\left(\text{ξ},\eta_{-1}\right)+\frac{{\hat{v}}_{1}\left(\text{ξ},\eta_{-1}\right)}{\sqrt{{\left|\eta_{-1}\right|}^{2}-|\text{ξ}{|}^{2}}}\right)=0,\forall \left|\text{ξ}\right|<\left|\eta_{-1}\right|\right\},\\ X^{\tau }=\left\{v=\left(\begin{array}{c} v_{o}\\ v_{1} \end{array}\right)\in \left.X\right|\left({\hat{v}}_{o}\left(\text{ξ},\eta_{-1}\right)-\frac{{\hat{v}}_{1}\left(\text{ξ},\eta_{-1}\right)}{\sqrt{{\left|\eta_{-1}\right|}^{2}-|\text{ξ}{|}^{2}}}\right)=0,\forall \left|\text{ξ}\right|<\left|\eta_{-1}\right|\right\},\\ X^{c}=\left\{v=\left(\begin{array}{c} v_{o}\\ v_{1} \end{array}\right)\in X|support\left(\begin{array}{c} {\hat{v}}_{o}\\ {\hat{v}}_{1} \end{array}\right)\subseteq \left\{\left|\eta_{-1}\right|\leq |\text{ξ}|\right\}\right\}=X^{\sigma }\cap X^{\tau }. \end{array}$$

To explicate the center space, *X*^*c*^, let $\lambda =\sqrt{{\left|\eta_{-1}\right|}^{2}-|\text{ξ}{|}^{2}}$. Over the Intersection $X^{c}=X^{\tau }\cap X^{\sigma }$,${\hat{v}}_{o}-\frac{{\hat{v}}_{1}}{\lambda }={\hat{v}}_{o}+\frac{{\hat{v}}_{1}}{\lambda }=0$. This implies that for $|\text{ξ}|<\left|\eta_{-1}\right|$,${\hat{v}}_{o}={\hat{v}}_{1}=0$. Therefore, non-trivial solutions of the Cauchy initial value problem require the $support\left(\begin{array}{c} {\hat{v}}_{o}\\ {\hat{v}}_{1} \end{array}\right)$ to be a subset of $\left\{\left|\eta_{-1}\right|\leq |\text{ξ}|\right\}$.

These three subdomains determine the corresponding three types of ultrahyperbolic wave equation solutions^22^:

1. For constraints in the central stable space, $v=\left(\begin{array}{l} v_{o}\\ v_{1} \end{array}\right)\in X^{\sigma }$, the Cauchy initial value problem has a *unique solution u∈X*, for all $\kappa_{1}=t\in {\mathbb{R}}^{+}$;
2. For constraints in the central unstable space, $v=\left(\begin{array}{l} v_{o}\\ v_{1} \end{array}\right)\in X^{\tau }$, the Cauchy initial value problem has a *unique solution u∈X*, only for all $\kappa_{1}=t\in {\mathbb{R}}^{-}$; and
3. For constraints in the central space, $v=\left(\begin{array}{l} v_{o}\\ v_{1} \end{array}\right)\in X^{c}$, the Cauchy initial value problem has a *unique global solution u∈X*, for all $\eta_{1}=t\in \mathbb{R}$.

In the case of periodic spatial and temporal domains, we require $|\xi {|}^{2}\equiv |\eta {|}^{2}$, where $\text{ξ}\in {\mathbb{Z}}^{d_{s}}$ and $\eta \in {\mathbb{Z}}^{d_{t}}$. The solutions are hence quite restrictive. If we instead consider the domain $x\in {\left[-1,1\right]}^{d_{s}}$ but $\kappa =\left(\kappa_{1},\kappa_{-1}\right)\in {\mathbb{R}}^{d_{t}}$, which allows for non-integer temporal frequencies, then we can obtain a wider spectrum of solutions.

Indeed, extending the Fourier transform solution from the case of periodic temporal domain, one can verify that linear combinations of solutions of the form:

(6.24) $$e^{2\pi i\langle \eta ,\kappa \rangle }\times e^{2\pi i\langle x,\text{ξ}\rangle },|\eta {|}^{2}=|\text{ξ}{|}^{2},$$

still solve the wave equation, since

(6.25) $$\Delta_{\kappa }\left(e^{2\pi i\langle \eta ,\kappa \rangle }\times e^{2\pi i\langle x,\text{ξ}\rangle }\right)=-4\pi^{2}|\eta {|}^{2}=-4\pi^{2}|\text{ξ}{|}^{2}=\Delta_{\text{ξ}}\left(e^{2\pi i\langle \eta ,\kappa \rangle }\times e^{2\pi i\langle x,\text{ξ}\rangle }\right).$$

The Cauchy initial value problem is well-posed when the solutions of this form lie in the center space *X*^*c*^ since ${\left|\eta_{-1}\right|}^{2}\leq {\left|\kappa_{1}\right|}^{2}+{\left|\eta_{-1}\right|}^{2}=\;|\eta {|}^{2}=\;|\text{ξ}{|}^{2}$, implying that $support\left(\begin{array}{c} {\hat{v}}_{o}\\ {\hat{v}}_{1} \end{array}\right)\subseteq \left\{\left|\eta_{-1}\right|\leq \;|\text{ξ}|\right\}$.

Conversely, global well-posed solutions given initial conditions $\left(v_{0},v_{1}\right)\in X^{c}$ can be computed as follows. First, we define the solution Fourier transform as

(6.26) $$\begin{array}{l} \hat{u}\left(\text{ξ},\kappa_{1},\eta_{-1}\right)=\cos\left(\kappa_{1}\sqrt{|\text{ξ}{|}^{2}-{\left|\eta_{-1}\right|}^{2}}\right)\times {\hat{v}}_{o}\left(\text{ξ},\eta_{-1}\right)\\ \;\;\;\;\;+\frac{\sin\left(\kappa_{1}\sqrt{|\text{ξ}{|}^{2}-{\left|\eta_{-1}\right|}^{2}}\right)}{\sqrt{|\text{ξ}{|}^{2}-{\left|\eta_{-1}\right|}^{2}}}{\hat{v}}_{1}\left(\text{ξ},\eta_{-1}\right). \end{array}$$

Note that since $v_{0}$, $v_{1}\in X^{c}$, we know that $\sqrt{|\text{ξ}{|}^{2}-{\left|\eta_{-1}\right|}^{2}}\in \mathbb{R}$; hence,

(6.27) $$\begin{array}{l} \left|\cos\left(\kappa_{1}\sqrt{|\text{ξ}{|}^{2}-{\left|\eta_{-1}\right|}^{2}}\right)\right|\leq 1\text{ and}\\ \;\frac{\sin\left(\kappa_{1}\sqrt{|\text{ξ}{|}^{2}-{\left|\eta_{-1}\right|}^{2}}\right)}{\sqrt{|\text{ξ}{|}^{2}-{\left|\eta_{-1}\right|}^{2}}}\leq \kappa_{1}, \end{array}$$

which implies that

(6.28) $$\begin{array}{l} {\left\|\hat{u}\left(\text{ξ},\kappa_{1},\eta_{-1}\right)\right\|}_{L^{2}}\leq {\left\|{\hat{v}}_{o}\left(\text{ξ},\eta_{-1}\right)\right\|}_{L^{2}}+\kappa_{1}{\left\|{\hat{v}}_{1}\left(\text{ξ},\eta_{-1}\right)\right\|}_{L^{2}}\\ \;\;\;\;\;\;={\left\|v_{0}\right\|}_{L^{2}}+\kappa_{1}{\left\|v_{1}\right\|}_{L^{2}}, \end{array}$$

where the last equality follows from Plancherel’s Theorem. Therefore, we may invert the Fourier transform to obtain an analytic solution for $u\left(x,\kappa_{1},\kappa_{-1}\right)$

(6.29) $$u\left(x,\kappa_{1},\kappa_{-1}\right):=\sum_{\xi \in {\mathbb{Z}}^{d_{s}}}\int_{{\mathbb{R}}^{d_{t}-1}}\hat{u}\left(\text{ξ},\kappa_{1},\eta_{-1}\right)\times e^{2\pi i\langle \eta_{-1},\kappa_{-1}\rangle }\times e^{2\pi i\langle x,\text{ξ}\rangle }d\eta_{-1}.$$

The special solutions

(6.30) $$u(x,\kappa )=\underset{\left\{\begin{array}{c} {\text{ξ}}_{m},\eta_{m}\\ {\left|{\text{ξ}}_{m}\right|}^{2}=\;{\left|\eta_{m}\right|}^{2} \end{array}\right\}}{\underset{\;}{\sum_{m=1}^{M}\;}}C_{m}\times e^{2\pi i\langle \eta_{m},\kappa \rangle }\times e^{2\pi i\langle x,{\text{ξ}}_{m}\rangle }$$

are just instances of the general solution formula, i.e., when only finitely many coefficients in the infinite sum are non-trivial.

Several applications showing Mathematica® demonstrations of the dynamics of the wave equation solutions in spacekime are shown in the appendix and life demonstrations are available on our dedicated web server (https://socr.umich.edu/TCIU/HTMLs/Chapter3_Wave_Equation_in_Spacekime.html).

## 7. Conclusion and discussion

In multiple time dimensions, the ultrahyperbolic wave equation, subject to Cauchy initial value constraints of co-dimension one, represents given data on dimension one less than the total dimension of the complete spacetime manifold. Without any additional constraints, this problem is ill-posed, which means that without additional information, or data, stable global solutions do not exist. However, by adding additional constraints, the Cauchy data problem has unique and stable solutions. Thus, the initial data and the nonlocal contains completely determine the behavior (data prediction, state forecasting) at all other times. This deterministic forecasting ensures that small perturbations in the specification of the initial data lead to proportionally small and controllable perturbations in the potential function (ultrahyperbolic wave equation solution).

The second-order ultrahyperbolic wave equation with Cauchy initial data restricts the potential function and its first normal derivative at each point on the co-dimension 1 hypersurface. Without the additional non-local constraints, the Cauchy data problem is not well-posed and the existence, uniqueness and stability of any solutions are not guaranteed. Even if such solutions exist, their practical predictability will be poor, which suggests that causal relations between inputs and outputs are unstable in such conditions. However, imposing additional nonlocal constraints on the initial data ensures the well-posedness of the Cauchy data problem.

These non-local constraints lead to the existence of stable and global solutions, which suggests that data science problems in spacekime would permit reliable, reproducible and stable forecasts. The specific (spectral) non-local constraints can be described in terms of the Fourier transforms of the initial data $\hat{f}\left(\xi ,\eta_{-1}\right)=ℱ\left(f\left(x,\kappa_{-1}\right)\right)\left(\xi ,\eta_{-1}\right)$ and $\hat{g}\left(\xi ,\eta_{-1}\right)=ℱ\left(g\left(x,\kappa_{-1}\right)\right)\left(\xi ,\eta_{-1}\right)$. These conditions restrict the spectral domains of the Fourier transform $\hat{f}$ and $\hat{g}$ to $\|\xi {\|}^{2}\geq {\left\|\kappa_{-1}\right\|}^{2}$.

This restriction leads naturally to the corresponding spacetime formulations of the Cauchy initial value problem via the inverse Fourier transform $f\left(x,\kappa_{-1}\right)={ℱ}^{-1}\left(\hat{f}\left(\xi ,\eta_{-1}\right)\right)\left(x,\kappa_{-1}\right)$ and $g\left(x,\kappa_{-1}\right)={ℱ}^{-1}\left(\hat{g}\left(\xi ,\eta_{-1}\right)\right)\left(x,\kappa_{-1}\right)$, indicating the more intuitive restrictions on the initial data that guarantee the problem well-posedness and stability of the solution.

Note that this constraint is non-local and formulates nontrivial associations between the values of the potential function solution at different spacekime points on the lower-dimensional hypersurface.

In the 4D Minkowski spacetime, utilizing only the kime-magnitude (time) of the complex time-like variables, uniqueness, stability, and determinism of the solution at any given time ensures the same is true at all other times.

The Cauchy data problem with proper constraints guarantees the same behavior in spacekime (with 2D complex time), i.e., the solution is still stable given initial data on a hypersurface of co-dimension one. However, in complex time, the hypersurface mixes spatial and temporal dimension constraints that are non-local, whereas in spacetime (1D time) the hypersurface is defined only using the spatial dimensions. In spacekime, the initial data may also be defined in general on a hypersurface of co-dimension 2, i.e., instead of fixing the initial conditions at $\kappa_{1}=0$, it may be specified purely on spacelike hypersurfaces $\left(\kappa_{1},\kappa_{2}\right)=(0,0)$.

The general stability of the Cauchy initial value problem in higher co-dimensions is intractable, since too little information about the initial state is provided to deterministically predict the (global) solution and explicate the dynamics of the potential function evolution in kime. In statistics and data science, this problem is similar to solving underdetermined linear modeling problems, where manipulating one or more of the degrees of freedom may drastically alter the model-forecasts. In physics, this situation is analogous to specifying the initial conditions of a field by only anchoring one or two of the spatial dimensions, which in general is insufficient to determine the field evolution over all times.

Local spacetime constraints are insufficient to guarantee a unique global solution of the general Cauchy data problem. No matter how much initial data is given on the initial hypersurface, e.g., specify the potential function value, and its derivatives in the *κ*_1_ and *κ*_2_ temporal directions, along with any number of higher order derivatives, the solution would not be unique. Therefore, the general ultrahyperbolic wave equation problem with Cauchy initial data in higher co-dimension (2+) does not permit global unique solutions without additional nonlocal constraints. However, an alternative problem formulation, where the Cauchy initial data problem is formulated locally over a contiguous neighborhood, guarantees that when a solution is known to exist, it will be global and unique over the entire spacekime.^21^

## Supplementary Material

supplementary materials

## Figures and Tables

**Table 1:** Examples of important partial differential equations of different types.

PDE name	Equation	Classification
Transport equation^15^	$\frac{\partial u}{\partial x}+c\frac{\partial u}{\partial y}=0$, where *c* is constant	Linear first-order PDE
Eikonal equation^16^	${\left(\frac{\partial u}{\partial x}\right)}^{2}+{\left(\frac{\partial u}{\partial y}\right)}^{2}=v^{2}(x,y)\geq 0$	Nonlinear first-order PDE
Laplace equation^17^	$\frac{\partial^{2}u}{\partial x^{2}}+\frac{\partial^{2}u}{\partial y^{2}}=0$	Elliptic linear second-order PDE
Heat equation^18^	$\frac{\partial u}{\partial t}-k\frac{\partial^{2}u}{\partial x^{2}}=f(x)$, where *k* is the thermal diffusivity	Parabolic linear second-order PDE, nonhomogeneous since the right hand side is a non-trivial function
Spacetime wave equation^19^	$\frac{\partial^{2}u}{\partial t^{2}}-c^{2}\frac{\partial^{2}u}{\partial x^{2}}=0$, where *c* is the speed of light	Hyperbolic linear second-order PDE in spacetime (*x, t*)
Spacekime wave equation^7^	$\frac{\partial^{2}u}{\partial \kappa_{1}^{2}}+\frac{\partial^{2}u}{\partial \kappa_{2}^{2}}-c^{2}\left(\frac{\partial^{2}u}{\partial x^{2}}+\frac{\partial^{2}u}{\partial y^{2}}+\frac{\partial^{2}u}{\partial z^{2}}\right)=0$, where *c* is the speed of light	Ultrahyperbolic linear second-order PDE, with respect to multidimensional time (*κ*_1_*, κ*_2_)
Navier–Stokes equations^14^	$\underset{\text{intertia}}{\underset{︸}{\underset{\text{variation }}{\underset{︸}{\frac{\partial u}{dt}}}+\underset{\text{convection }}{\underset{︸}{(u\cdot \nabla )u}}}}-\underset{\text{divergence stress}}{\underset{︸}{\underset{\text{diffusion}}{\underset{︸}{v\nabla^{2}u}}=\underset{\text{internal source}}{\underset{︸}{-\nabla \omega }}}}+\underset{\text{external source }}{\underset{︸}{g}}$	The convective form of the incompressible Navier–Stokes equations (may be of different classification types) are used to model the motion of viscous fluids. Notation: *u* is the fluid velocity vector, *ω* is the fluid internal pressure relative to the fluid density, ν is the kinematic viscosity, ∇ and ∇^2^ are the gradient and the Laplacian operators, and *g* is an external force

**Table 2:** Examples of specific boundary conditions and their corresponding wave equation solutions.

Boundary condition	Solution
${\left.u\right|}_{|\kappa |=1}=e^{\left(3ix_{1}-4ix_{2}\right)}\cdot g$	$u(t,\theta ,\varphi ,x)=g\cdot \frac{J_{\frac{1}{2}}(5t)}{\sqrt{t}\times J_{\frac{1}{2}}(5)}e^{\left(3ix_{1}-4ix_{2}\right)}$
${\left.u\right|}_{|\kappa |=1}=e^{\left(3ix_{1}-4ix_{2}\right)}\cdot \cos\theta \cdot g$	$u(t,\theta ,\varphi ,x)=g\cdot \frac{J_{\frac{3}{2}}(5t)}{\sqrt{t}\times J_{\frac{3}{2}}(5)}e^{\left(3ix_{1}-4ix_{2}\right)}\cdot \cos(\theta )$
${\left.u\right|}_{|\kappa |=1}=e^{\left(3ix_{1}-4ix_{2}\right)}\cdot \left(\sin\theta \cdot e^{i\varphi }\right)g$	$u(t,\theta ,\varphi ,x)=g\cdot \frac{J_{\frac{3}{2}}\left(5t\right)}{\sqrt{t}\times J_{\frac{3}{2}}(5)}e^{\left(3ix_{1}-4ix_{2}\right)}\cdot \sin(\theta )\cdot e^{i\varphi }$

## Box I.

(6.8)
∂κ1E[u]=12∂κ1∫∂κ1u2+∇xu2−∇κ−1u2dxdκ−1=12∫2∂κ1u×∂κ12u+∇xu×∇x∂κ1(u)−∇κ−1u×∇κ−1∂κ1(u)dxdκ−1=∫∫∂κ1u×∂κ12udx+∫∇xu×∇x∂κ1(u)−∇κ−1u×∇κ−1∂κ1(u)dxdκ−1=∫∫∂κ1u×∂κ12udxdκ−1+∫∇xu⋅∇x∂κ1udxdκ−1−∫∇κ−1u×∇κ−1∂κ1(u)dκ−1dx=∫∫∂κ1u×∂κ12udxdκ−1+∫∇xu⋅∇x∂κ1udxdκ−1−∫∇κ−1u×∇κ−1∂κ1(u)dκ−1dx=∫∫∂κ1u×∂κ12udxdκ−1+∫∫∂Dt−1∂κ1u⋅∇xu⋅n^︸0dxdκ−1−∫∇x2u∂κ1udxdκ−1−∫∫∂Dt−1∂κ1u⋅∇κ−1u⋅n^︸0dκ−1dx+∫∇κ−12u⋅∂κ1udxdκ−1=∫∫∂κ1u×∂κ12udxdκ−1−∫∇x2u∂κ1udxdκ−1+∫∇κ−12u⋅∂κ1udxdκ−1=∫∫∂κ1u⋅∂κ12u−∇x2u+∇κ−12udκ−1dx=0.

## Footnotes

1 Laplacian with Dirichlet boundary conditions is self-adjoint.
