# Supplementary material for: Determinism, well-posedness, and applications of the ultrahyperbolic wave equation in spacekime
Source: Partial Differ Equ Appl Math. Author manuscript; Available in PMC 2023 Jun 1. (PMC9494226; doi:10.1016/j.padiff.2022.100280)
Supplement: supplementary materials [file NIHMS1832591-supplement-supplementary_materials.docx]

**Supplementary Materials (Manuscript Appendix)**

*Determinism, Well-posedness, and Applications of the Ultrahyperbolic*

*Wave Equation in Spacekime*

Yuxin Wang ^1,4^, Yueyang Shen ^2,4^, Daxuan Deng ^3,4^, Ivo D. Dinov ^4,5^ *

^1^ Department of Mathematics

^2^ Electrical Computer Engineering Division

^3^ Department of Statistics

^4^ Statistics Online Computational Resource, Department of Health Behavior and Biological Sciences

^5^ Department of Computational Medicine and Bioinformatics

University of Michigan, Ann Arbor, MI 48109, USA

*** Contact:**

Ivo D. Dinov

Statistics Online Computational Resource

University of Michigan

426 N. Ingalls Str.

Ann Arbor, MI 48109

Email: [statistics@umich.edu](mailto:statistics@umich.edu) | URL: <https://umich.edu/~dinov> | Tel: 1 + 734-764-5556

1. **Derivation of the examples**

**Example 1.** Consider the special case of the ultrahyperbolic wave equation in spherical time coordinates under the following boundary condition

$$u\left. \right|_{\left| \boldsymbol{\kappa} \right|=1}=e^{\left( 3ix_{1}-4ix_{2} \right)}\cdot g ,$$

where $g$ is some real constant that is independent of $\theta,\varphi,x$. We will follow the recipe specified above

$$\left\langle e^{i\left\langle\boldsymbol{\xi}\boldsymbol{,x} \right\rangle}F_{\boldsymbol{\xi}}\left( 1,\theta,\varphi\right),f\left( \boldsymbol{\kappa,x} \right)\left. \right|_{\left| \boldsymbol{\kappa} \right|=1} \right\rangle_{L^{2}}=\int_{L^{2}} e^{i\left\langle\boldsymbol{\xi}\boldsymbol{,x} \right\rangle}F_{\boldsymbol{\xi}}\left( 1,\theta,\varphi\right)\underset{complex conjugate}{\underbrace{\bar{f\left( \boldsymbol{\kappa,x} \right)\left. \right|_{\left| \boldsymbol{\kappa} \right|=1}}}}\sin\left( \theta\right) d\theta d\varphi d\boldsymbol{x}\boldsymbol{=}$$

$$\int_{-\pi}^{\pi} \int_{-\pi}^{\pi} e^{i\xi_{1}x_{1}}e^{i\xi_{2}x_{2}}\int_{0}^{2\pi} \int_{0}^{\pi} J_{\left( n+\frac{1}{2} \right)}\left( \left| \boldsymbol{\xi} \right| \right)P_{n}^{m}\left( \cos\theta\right)e^{im\varphi}e^{\left( -3ix_{1}+4ix_{2} \right)}\cdot g\cdot\sin\left( \theta\right) d\theta d\varphi dx_{1}dx_{2}=$$

$$g J_{\left( n+\frac{1}{2} \right)}\left( \left| \boldsymbol{\xi} \right| \right)\left( \int_{-\pi}^{\pi} \int_{-\pi}^{\pi} e^{i\xi_{1}x_{1}}e^{i\xi_{2}x_{2}}\cdot e^{\left( -3ix_{1}+4ix_{2} \right)}dx_{1}dx_{2} \right)\left( \int_{0}^{2\pi} \int_{0}^{\pi} P_{n}^{m}\left( \cos\theta\right)e^{im\varphi}\sin\left( \theta\right)d\theta d\varphi\right) .$$

Note that

$$\int_{-\pi}^{\pi} e^{i\xi_{1}x_{1}}\cdot e^{-3ix_{1}}dx_{1}=\int_{0}^{2\pi} e^{i\xi_{1}x_{1}}\cdot e^{-3ix_{1}}dx_{1}=2\pi\delta_{\xi_{1},3}=\left\{ \begin{aligned} 2\pi, \xi_{1}=3 \\ 0, \xi_{1}\neq3 \end{aligned} \right. .$$

Thus,

$$\frac{\left\langle e^{i\left\langle\boldsymbol{\xi}\boldsymbol{,x} \right\rangle}F_{\boldsymbol{\xi}}\left( 1,\theta,\varphi\right),f\left( \boldsymbol{\kappa,x} \right)\left. \right|_{\left| \boldsymbol{\kappa} \right|=1} \right\rangle_{L^{2}}}{gJ_{\left( n+\frac{1}{2} \right)}\left( \left| \boldsymbol{\xi} \right| \right)}=\left( 2\pi\delta_{\xi_{1},3} \right)\left( 2\pi\delta_{\xi_{2},-4} \right)\left( \int_{0}^{2\pi} e^{im\varphi}d\varphi\right)\left( \int_{0}^{\pi} P_{n}^{m}\left( \cos\theta\right)\cdot d\theta\right)=$$

$$\left( 2\pi\delta_{\xi_{1},3} \right)\left( 2\pi\delta_{\xi_{2},-4} \right)\left( 2\pi\delta_{m,0} \right)\underset{\left( \int_{-1}^{1} P_{n}\left( x \right)\cdot dx \right)=\left( 2\delta_{n,0} \right)}{\underbrace{\left( \int_{0}^{\pi} P_{n}^{0}\left( \cos\theta\right)\cdot\sin\left( \theta\right)d\theta\right)}}=16\pi^{3}\delta_{\xi_{1},3}\delta_{\xi_{2},-4}\delta_{m,0}\delta_{n,0} .$$

The denominator can be taken care of via:

$$\left\| e^{i\left\langle\boldsymbol{\xi}\boldsymbol{,x} \right\rangle}F_{\boldsymbol{\xi}}\left( 1,\theta,\varphi\right) \right\|_{L^{2}}\boldsymbol{=}$$

$$\int_{L^{2}} e^{i\left\langle\boldsymbol{\xi}\boldsymbol{,x} \right\rangle}P_{n}^{m}\left( \cos\theta\right)e^{im\varphi}J_{\left( n+\frac{1}{2} \right)}\left( \left| \boldsymbol{\xi} \right| \right)\underset{complex conjugate}{\underbrace{e^{-i\left\langle\boldsymbol{\xi}\boldsymbol{,x} \right\rangle}P_{n}^{m}\left( \cos\theta\right)e^{-im\varphi}J_{\left( n+\frac{1}{2} \right)}\left( \left| \boldsymbol{\xi} \right| \right)}}\sin\left( \theta\right) d\theta d\varphi d\boldsymbol{x}=$$

$$8\pi^{3}\int_{0}^{\pi} P_{n}^{m}\left( \cos\theta\right)P_{n}^{m}\left( \cos\theta\right)\cdot\sin\left( \theta\right)\left( J_{\left( n+\frac{1}{2} \right)}\left( \left| \boldsymbol{\xi} \right| \right) \right)^{2}d\theta=8\pi^{3}\left( J_{\left( n+\frac{1}{2} \right)}\left( \left| \boldsymbol{\xi} \right| \right) \right)^{2}\int_{-1}^{1} P_{n}^{m}\left( x \right)P_{n}^{m}\left( x \right)\cdot dx ,$$

where the associated Legendre polynomials satisfy orthogonality conditions

$$\int_{-1}^{1} P_{k}^{m}P_{l}^{m}dx=\frac{2\left( l+m \right)!}{\left( 2l+1 \right)\left( l-m \right)!}\delta_{k,l} .$$

Thus, we can write the scalar factors as

$$C_{\xi,n,m}=\frac{\left\langle e^{i\left\langle\boldsymbol{\xi}\boldsymbol{,x} \right\rangle}F_{\boldsymbol{\xi}}\left( 1,\theta,\varphi\right),f\left( \boldsymbol{\kappa,x} \right)\left. \right|_{\left| \boldsymbol{\kappa} \right|=1} \right\rangle_{L^{2}}}{\left\langle e^{i\left\langle\boldsymbol{\xi}\boldsymbol{,x} \right\rangle}F_{\boldsymbol{\xi}}\left( 1,\theta,\varphi\right),e^{i\left\langle\boldsymbol{\xi}\boldsymbol{,x} \right\rangle}F_{\boldsymbol{\xi}}\left( 1,\theta,\varphi\right) \right\rangle_{L^{2}}}=\frac{16\pi^{3}\delta_{\xi_{1},3}\delta_{\xi_{2},-4}\delta_{m,0}\delta_{n,0}}{8\pi^{3}\left( J_{\left( n+\frac{1}{2} \right)}\left( \left| \boldsymbol{\xi} \right| \right) \right)^{2}\frac{2\left( n+m \right)!}{\left( 2n+1 \right)\left( n-m \right)!}}g .$$

Then, the solution to the boundary value problem satisfies

$$u\left( t,\theta,\varphi,x \right)=$$

$$\sum_{n=0}^{\infty} \sum_{m=0}^{n} \sum_{\boldsymbol{\xi}\in\mathbb{Z}^{2}} g\frac{16\pi^{3}\delta_{\xi_{1},3}\delta_{\xi_{2},-4}\delta_{m,0}\delta_{n,0}}{8\pi^{3}\left( J_{\left( n+\frac{1}{2} \right)}\left( \left| \boldsymbol{\xi} \right| \right) \right)^{2}\frac{2\left( n+m \right)!}{\left( 2n+1 \right)\left( n-m \right)!}}\cdot e^{i\left\langle\boldsymbol{\xi}\boldsymbol{,x} \right\rangle}\frac{1}{\sqrt{t}} J_{\left( n+\frac{1}{2} \right)}\left( \left| \boldsymbol{\xi} \right|t \right)P_{n}^{m}\left( \cos\left( \theta\right) \right)e^{im\varphi}=$$

$$g \frac{J_{\frac{1}{2}}\left( 5t \right)}{\sqrt{t} J_{\frac{1}{2}}\left( 5 \right)}e^{\left( 3ix_{1}-4ix_{2} \right)} .$$

**Example 2.** In the second example, consider the special case where the boundary condition is

$$u\left. \right|_{\left| \boldsymbol{\kappa} \right|=1}=e^{\left( 3ix_{1}-4ix_{2} \right)}\cdot\cos\theta\cdot g ,$$

$$\frac{\left\langle e^{i\left\langle\boldsymbol{\xi}\boldsymbol{,x} \right\rangle}F_{\boldsymbol{\xi}}\left( 1,\theta,\varphi\right),f\left( \boldsymbol{\kappa,x} \right)\left. \right|_{\left| \boldsymbol{\kappa} \right|=1} \right\rangle_{L^{2}}}{gJ_{\left( n+\frac{1}{2} \right)}\left( \left| \boldsymbol{\xi} \right| \right)}=\left( 2\pi\delta_{\xi_{1},3} \right)\left( 2\pi\delta_{\xi_{2},-4} \right)\left( 2\pi\delta_{m,0} \right)\left( \int_{0}^{\pi} P_{n}^{0}\left( \cos\theta\right)\cdot\sin\left( \theta\right)\cdot\cos\left( \theta\right)d\theta\right)=$$

$$8\pi^{3}\delta_{\xi_{1},3}\delta_{\xi_{2},-4}\delta_{m,0}\frac{2\sin\left( n\pi\right)}{\left( -2-n+n^{2} \right)\pi}=8\pi^{3}\delta_{\xi_{1},3}\delta_{\xi_{2},-4}\delta_{m,0}\left( \frac{2}{3}\delta_{n,1} \right) .$$

Thus,

$$\left\| e^{i\left\langle\boldsymbol{\xi}\boldsymbol{,x} \right\rangle}F_{\boldsymbol{\xi}}\left( 1,\theta,\phi\right) \right\|_{L^{2}}=8\pi^{3}\int_{-1}^{1} P_{n}^{m}\left( x \right)P_{n}^{m}\left( x \right)\cdot dx ,$$

$$C_{\xi,n,m}=\frac{\left\langle e^{i\left\langle\boldsymbol{\xi}\boldsymbol{,x} \right\rangle}F_{\boldsymbol{\xi}}\left( 1,\theta,\varphi\right),f\left( \boldsymbol{\kappa,x} \right)\left. \right|_{\left| \boldsymbol{\kappa} \right|=1} \right\rangle_{L^{2}}}{\left\langle e^{i\left\langle\boldsymbol{\xi}\boldsymbol{,x} \right\rangle}F_{\boldsymbol{\xi}}\left( 1,\theta,\varphi\right),e^{i\left\langle\boldsymbol{\xi}\boldsymbol{,x} \right\rangle}F_{\boldsymbol{\xi}}\left( 1,\theta,\varphi\right) \right\rangle_{L^{2}}}=\frac{\delta_{\xi_{1},3}\delta_{\xi_{2},-4}\delta_{m,0}\left( \frac{2}{3}\delta_{n,1} \right)}{\left( J_{\left( n+\frac{1}{2} \right)}\left( \left| \boldsymbol{\xi} \right| \right) \right)^{2}\frac{2\left( n+m \right)!}{\left( 2n+1 \right)\left( n-m \right)!}}g ,$$

$$u\left( t,\theta,\varphi,x \right)=$$

$$\sum_{n=0}^{\infty} \sum_{m=0}^{n} \sum_{\boldsymbol{\xi}\in\mathbb{Z}^{2}} \left( g\frac{\delta_{\xi_{1},3}\delta_{\xi_{2},-4}\delta_{m,0}\left( \frac{2}{3}\delta_{n,1} \right)}{\left( J_{\left( n+\frac{1}{2} \right)}\left( \left| \boldsymbol{\xi} \right| \right) \right)^{2}\frac{2\left( n+m \right)!}{\left( 2n+1 \right)\left( n-m \right)!}}\cdot e^{i\left\langle\boldsymbol{\xi}\boldsymbol{,x} \right\rangle}\cdot\frac{1}{\sqrt{t}} \cdot J_{\left( n+\frac{1}{2} \right)}\left( \left| \boldsymbol{\xi} \right|t \right)\cdot P_{n}^{m}\left( \cos\left( \theta\right) \right)\cdot e^{im\varphi} \right)=$$

$$\frac{\left( \frac{2}{3} \right)}{\left( J_{\left( \frac{3}{2} \right)}\left( 5 \right) \right)^{2}\frac{2}{3}}\cdot e^{\left( 3ix_{1}-4ix_{2} \right)}\frac{1}{\sqrt{t}} J_{\left( \frac{3}{2} \right)}\left( 5t \right)P_{1}^{0}\left( \cos\left( \theta\right) \right)g=\frac{J_{\frac{3}{2}}\left( 5t \right)}{\sqrt{t}\times J_{\frac{3}{2}}\left( 5 \right)}e^{\left( 3ix_{1}-4ix_{2} \right)}\cdot\cos\left( \theta\right)\cdot g .$$

**Example 3**. Next, consider the special case where the boundary condition is

$$u\left. \right|_{\left| \boldsymbol{\kappa} \right|=1}=e^{3ix_{1}-4ix_{2}}\cdot(\sin\theta\cdot e^{i\varphi})g ,$$

$$\boldsymbol{x}\in\left[ -\pi,\pi\right)^{2}, \theta\in\left[ -\frac{\pi}{2},\frac{\pi}{2} \right], \varphi\in\left[ -\pi,\pi\right).$$

The solution can be derived via

$$\frac{\left\langle e^{i\left\langle\boldsymbol{\xi}\boldsymbol{,x} \right\rangle}F_{\boldsymbol{\xi}}\left( 1,\theta,\phi\right),f\left( \boldsymbol{\kappa,x} \right)\left. \right|_{\left| \boldsymbol{\kappa} \right|=1} \right\rangle_{L^{2}}}{g J_{\left( n+\frac{1}{2} \right)}\left( \left| \boldsymbol{\xi} \right| \right)}=$$

$$\left( 2\pi\delta_{\xi_{1},3} \right)\left( 2\pi\delta_{\xi_{2},-4} \right)\int_{0}^{2\pi} e^{\left( im\varphi-\varphi\right)}d\varphi\left( \int_{0}^{\pi} P_{n}^{m}\left( \cos\theta\right)\cdot\sin\left( \theta\right)\cdot\sin\left( \theta\right)d\theta\right)=$$

$$8\pi^{3}\delta_{\xi_{1},3}\delta_{\xi_{2},-4}\delta_{m,1}\left( \int_{0}^{\pi} P_{n}^{1}\left( \cos\theta\right)\cdot\underset{\sin\left( \theta\right)}{\underbrace{P_{1}^{1}\left( \cos\theta\right)}}\cdot\sin\left( \theta\right)d\theta\right) .$$

We again utilize the relation

$$\int_{-1}^{1} P_{k}^{m}\left( \cos\theta\right)P_{l}^{m}\left( \cos\theta\right)\sin\left( \theta\right)d\theta=\frac{2\left( l+m \right)!}{\left( 2l+1 \right)\left( l-m \right)!}\delta_{k,l}$$

$$\Longrightarrow\frac{\left\langle e^{i\left\langle\boldsymbol{\xi}\boldsymbol{,x} \right\rangle}F_{\boldsymbol{\xi}}\left( 1,\theta,\phi\right),f\left( \boldsymbol{\kappa,x} \right)\left. \right|_{\left| \boldsymbol{\kappa} \right|=1} \right\rangle_{L^{2}}}{g J_{\left( n+\frac{1}{2} \right)}\left( \left| \boldsymbol{\xi} \right| \right)}=\frac{2\left( 1+n \right)!}{\left( 2+1 \right)}\delta_{n,1} .$$

The scalar coefficient can be similarly written as

$$C_{\xi,n,m}=\frac{\left\langle e^{i\left\langle\boldsymbol{\xi}\boldsymbol{,x} \right\rangle}F_{\boldsymbol{\xi}}\left( 1,\theta,\varphi\right),f\left( \boldsymbol{\kappa,x} \right)\left. \right|_{\left| \boldsymbol{\kappa} \right|=1} \right\rangle_{L^{2}}}{\left\langle e^{i\left\langle\boldsymbol{\xi}\boldsymbol{,x} \right\rangle}F_{\boldsymbol{\xi}}\left( 1,\theta,\varphi\right),e^{i\left\langle\boldsymbol{\xi}\boldsymbol{,x} \right\rangle}F_{\boldsymbol{\xi}}\left( 1,\theta,\varphi\right) \right\rangle_{L^{2}}}=\frac{\delta_{\xi_{1},3}\delta_{\xi_{2},-4}\delta_{m,1}\frac{2\left( 1+n \right)!}{\left( 2+1 \right)}\delta_{n,1}}{\left( J_{\left( n+\frac{1}{2} \right)}\left( \left| \boldsymbol{\xi} \right| \right) \right)^{2}\frac{2\left( n+m \right)!}{\left( 2n+1 \right)\left( n-m \right)!}}g .$$

Therefore, the PDE solution can be explicated as

$$u\left( t,\theta,\varphi,x \right)=\sum_{n=0}^{\infty} \sum_{m=0}^{n} \sum_{\boldsymbol{\xi}\in\mathbb{Z}^{2}} \frac{\delta_{\xi_{1},3}\delta_{\xi_{2},-4}\delta_{m,1}\frac{2\left( 1+n \right)!}{\left( 2+1 \right)}\delta_{n,1}}{\left( J_{\left( n+\frac{1}{2} \right)}\left( \left| \boldsymbol{\xi} \right| \right) \right)^{2}\frac{2\left( n+m \right)!}{\left( 2n+1 \right)\left( n-m \right)!}}\cdot e^{i\left\langle\boldsymbol{\xi}\boldsymbol{,x} \right\rangle}\frac{1}{\sqrt{t}} J_{\left( n+\frac{1}{2} \right)}\left( \left| \boldsymbol{\xi} \right|t \right)P_{n}^{m}\left( \cos\left( \theta\right) \right)e^{im\varphi}g=$$

$$\frac{1}{\left( J_{\left( \frac{3}{2} \right)}\left( 5 \right) \right)^{2}}\cdot e^{\left( 3ix_{1}-4ix_{2} \right)}\frac{1}{\sqrt{t}} J_{\left( \frac{3}{2} \right)}\left( 5t \right)P_{1}^{1}\left( \cos\left( \theta\right) \right)e^{i\varphi}g=\frac{J_{\frac{3}{2}}\left( 5t \right)}{\sqrt{t}\times J_{\frac{3}{2}}\left( 5 \right)}e^{\left( 3ix_{1}-4ix_{2} \right)}\cdot sin\left( \theta\right)e^{i\varphi}\cdot g .$$

1. **The Mathematica notebook**

We attach the exported version of a Mathematica® Notebook implementing the derived solutions.

Spacekime Visualization of the Ultrahyperbolic Wave Equation using Mathematica

# Problem setup

One approach to understand wave dynamics in spacekime is to plot the basic separable solution. Recall the **5D wave equation**.

$$\Delta_{x}u=\Delta_{t}u,\mathrm{where}x=\left( x_{1},x_{2},x_{3} \right)\in\mathbb{R}^{3},t=\left( t_{1},t_{2} \right)\in\mathbb{R}^{2}.$$

The equation is essentially an ultrahyperbolic equation, and in general, if we do not impose a proper condition, the equation does not permit stable and unique and global solutions. One possible approach to resolve these instabilities of the potential functions is to impose Periodic Boundary conditions (PBC) and consider the corresponding base function solutions. In general, the basis takes the form

$$u\left( x,t \right)=e^{2\pi i\left( x_{1}\xi_{1}+x_{2}\xi_{2}+x_{3}\xi_{3}+t_{1}\eta_{1}+t_{2}\eta_{2} \right)},\xi_{1},\xi_{2},\xi_{3},\eta_{1},\eta_{2}\in\mathbb{Z}.$$

For the periodic boundary conditions to hold, we require that “multiples” of the spatial and temporal coordinates are integers. In the simplest scenario, we consider a “degenerated” hyperbolic equation to visualize the dynamics of wave equation under Periodic boundary conditions on the spatial dimension, a simpler case in 1 spatial dimension and one-time dimension is:

$$\Delta_{x}u=\Delta_{t}u,x\in\mathbb{R},t\in\mathbb{R},u\left( -1,t \right)=u\left( 1,t \right),u_{x}\left( -1,t \right)=u_{x}\left( 1,t \right),u\left( x,0 \right)=$$

$$\cos\left( \pi x \right), u_{t}\left( x,0 \right)=-\pi\sin\left( \pi x \right).$$

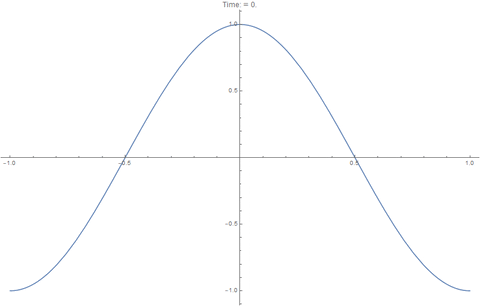


The previous animation can be generated via Mathematica®. Note that, once we have established the PBC conditions, the initial condition cannot be arbitrarily prescribed. PBC have to be

- Linear combinations of trigonometric sine function, and
- The frequency terms for time and space should be an integer multiple of $\frac{2\pi}{length of interval}$ . In this case it is $\pi$.

Here is the Mathematica code to model this solution (users may need to add a specific local export directory at the end).

eqn = Derivative[2, 0][u][x, t] == D[D[u[x, t], t], t];
(*The coefficient in Cosine must be a multiple of Pi*)
init1 = u[x, 0] == Cos[Pi*x];
init2 =
\!\(\*SuperscriptBox[\(u\),
TagBox[
RowBox[{"(",
RowBox[{"0", ",", "1"}], ")"}],
Derivative],
MultilineFunction->None]\)[x, 0] == -Pi*Sin[Pi*x];
(*Two initial conditions, Two boundary conditions*)
bon1 = u[-1, t] == u[1, t];
bon2 = (D[u[x, t], x] /. x -> -1) == (D[u[x, t], x] /. x -> 1);
usol = NDSolveValue[{eqn, init1, init2, bon1, bon2},
 u, {x, -1, 1}, {t, 0, 5}];
 fr = Table[
 Plot[usol[x, t], {x, -1, 1}, PlotLegends -> Automatic,
 PlotLabel -> (HoldForm["Time:"]) == t], {t, 0, 5, 0.2}];
Export["(*)", fr,
 "DisplayDurations" -> 0.3, "AnimationReptitions" -> 20,
 ImageSize -> 800];

Several alternative examples show the dynamics assuming same periodic boundary condition

$$u\left( x,0 \right)=\cos\left( \pi x \right),u_{x}\left( x,0 \right)=-4\pi\sin\left( \pi x \right) ,$$

$$u\left( x,0 \right)=\cos\left( \pi x \right)+\sin\left( 3\pi x \right),u_{x}\left( x,0 \right)=-\pi\sin\left( \pi x \right)+2\pi\cos\left( 3\pi x \right) ,$$

$$u\left( x,0 \right)=\cos\left( \pi x \right)+\sin\left( 3\pi x \right)+\cos\left( 5\pi x \right),u_{x}\left( x,0 \right)=-\pi\sin\left( \pi x \right)+2\pi\cos\left( 3\pi x \right)+\sin\left( 5\pi x \right) .$$

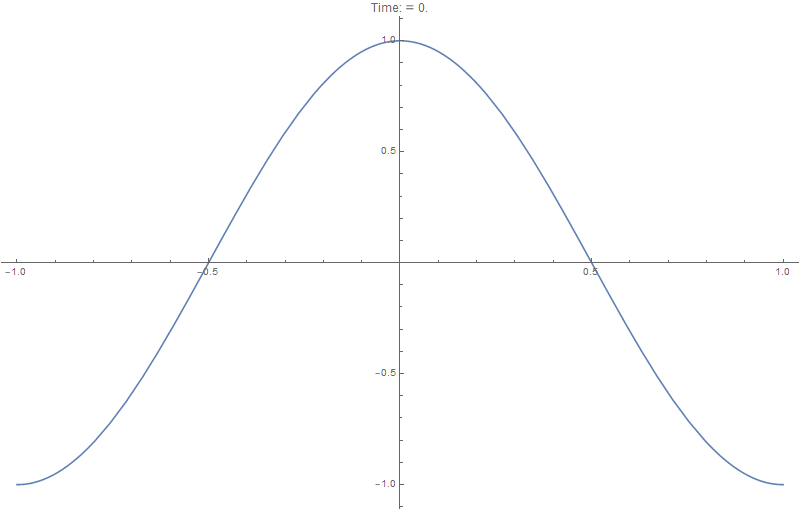

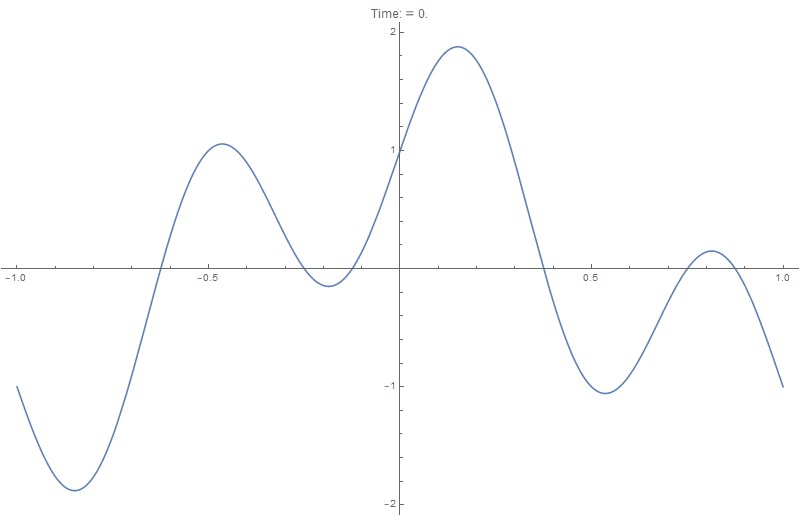

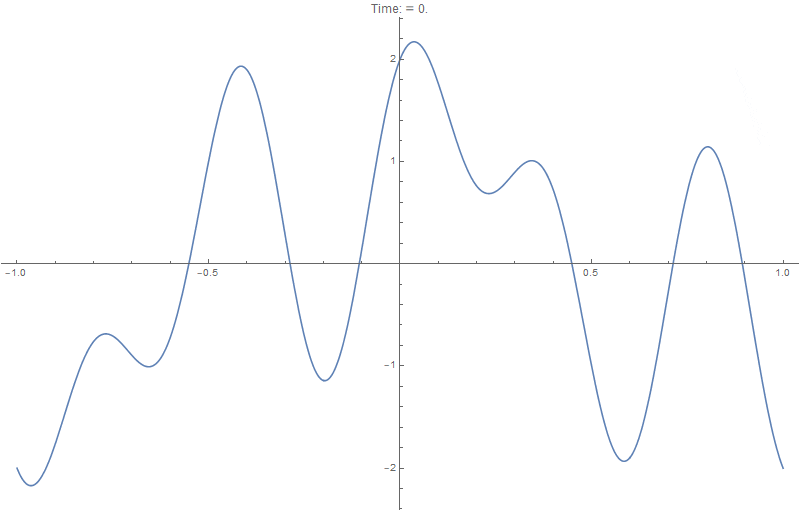


Next, we formalize a problem for the ultrahyperbolic equation with 2 time dimensions and 2 spatial dimensions, with periodic boundary conditions constrained on 2D spatial and 2D time dimensions.

$$\Delta_{x}u=\Delta_{t}u,\mathrm{where}x\in\left[ -\frac{1}{2},\frac{1}{2} \right]\times\left[ -\frac{1}{2},\frac{1}{2} \right]\subset\mathbb{R}^{2},t\in[0,5]\times[0,5]\subset\mathbb{R}^{2}$$

$$u\left( -\frac{1}{2},x_{2},t_{1},t_{2} \right)=u\left( \frac{1}{2},x_{2},t_{1},t_{2} \right),u_{x_{1}}\left( -\frac{1}{2},x_{2},t_{1},t_{2} \right)=u_{x_{1}}\left( \frac{1}{2},x_{2},t_{1},t_{2} \right),$$

$$u\left( x_{1},-\frac{1}{2},t_{1},t_{2} \right)=u\left( x_{1},\frac{1}{2},t_{1},t_{2} \right), u_{x_{2}}\left( x_{1},-\frac{1}{2},t_{1},t_{2} \right)=u_{x_{2}}\left( x_{1},\frac{1}{2},t_{1},t_{2} \right) ,$$

$$u\left( x_{1},x_{2},0,t_{2} \right)=u\left( x_{1},x_{2},5,t_{2} \right), u_{t_{1}}\left( x_{1},x_{2},0,t_{2} \right)=u_{t_{1}}\left( x_{1},x_{2},5,t_{2} \right) ,$$

$$u\left( x_{1},x_{2},t_{1},0 \right)=u\left( x_{1},x_{2},t_{1},5 \right), u_{t_{2}}\left( x_{1},x_{2},t_{1},0 \right)=u_{t_{2}}\left( x_{1},x_{2},t_{1},5 \right).$$

In polar kime coordinates, we have a slightly different formulation

$$\Delta_{x}u=\frac{\partial^{2}}{\partial r^{2}}u+\frac{1}{r}\frac{\partial}{\partial r}u+\frac{1}{r^{2}}\frac{\partial^{2}}{\partial\phi^{2}}u,\mathrm{where}\left( r,\phi\right)\in\left[ 0,5 \right]\times\left[ -\pi,\pi\right].$$

Instead of directly solving the polar kime problem, we transform the solution using Cartesian to polar coordinate transformations:

$$u\left( x,t \right)=e^{2\pi i\left( x_{1}\xi_{1}+x_{2}\xi_{2}+x_{3}\xi_{3}+\eta_{1}r\cos\phi+\eta_{2}r\sin\phi\right)}, \xi_{1},\xi_{2},\xi_{3},\eta_{1},\eta_{2}\in\mathbb{Z},$$

$$\left[ -1,1 \right]\times\left[ -1,1 \right]\times\underset{\mathrm{Cartesian}}{\underbrace{\left[ -5,5 \right]\times\left[ -5,5 \right]}} \Longrightarrow\left[ -1,1 \right]\times\left[ -1,1 \right]\times\underset{\mathrm{polar}}{\underbrace{\left[ 0,5 \right]\times\left( -\pi,\pi\right]}}.$$

However, the boundary condition is not necessarily determined by the boundary conditions in polar coordinates, but rather by the boundary conditions on the hypercube that “circumscribes” such a circle.

Strictly speaking, for the interval

$$[-1,1]^{ds} \mathrm{and} [-1,1]^{dt}$$

the solution should be

$$u\left( x,t \right)=e^{\pi i\left( x_{1}\xi_{1}+x_{2}\xi_{2}+x_{3}\xi_{3}+\eta_{1}r\cos\phi+\eta_{2}r\sin\phi\right)}, \xi_{1},\xi_{2},\xi_{3},\eta_{1},\eta_{2}\in\mathbb{Z}.$$

In the following animation we scale the solution by a factor of 2. That is,

$$u\left( x,t \right)=e^{2\pi i\left( x_{1}\xi_{1}+x_{2}\xi_{2}+x_{3}\xi_{3}+\eta_{1}r\cos\phi+\eta_{2}r\sin\phi\right)}, \xi_{1},\xi_{2},\xi_{3},\eta_{1},\eta_{2}\in\mathbb{Z} .$$

will still solve the periodic boundary condition on the $[-1,1]$ hypercube.

# Plane Waves (Independent kime dimensions)

##

## Result 1a: Wave Equation with Cylindrical Topology.

Let’s visualize the solution in terms of the kime phase coordinates. The setup is

$$\xi=(\xi_{1},\xi_{2},\xi_{3})=(-3,2,0),\eta=(\eta_{1},\eta_{2})=(-2,3),(x_{1},x_{2},t,\phi):[-1,1]\times[-1,1]\times[0,5]\times(-\pi,\pi)$$

**Note:** The rendering of this animation may take about 8-12 hours to complete.

eta = {-2, 3};
chsi = {-3, 2};
mp[\[Theta]_, x_, y_, t_] =
 Exp[2*Pi*I*t*
 Inner[Times, eta, {Cos[\[Theta]], Sin[\[Theta]]}, Plus]]*
 Exp[2*Pi*I*Inner[Times, chsi, {x, y}, Plus]];
fr = Table[
 Show[ContourPlot3D[
 Re[mp[\[Theta], x, y, 1]], {x, -1, 1}, {y, -1,
 1}, {\[Theta], -\[Pi], \[Pi]},
 RegionFunction -&gt;
 Function[{x, y, \[Theta]}, x^2 + y^2 &lt;= 1],
 Contours -&gt; {-0.5, 0, 0.5},
 ContourStyle -&gt; Opacity[0.1]],
 SliceContourPlot3D[Re[mp[\[Theta], x, y, 1]],
 x == -1, {x, -1, 1}, {y, -1,
 1}, {\[Theta], -\[Pi], \[Pi]}],
 SliceContourPlot3D[Re[mp[\[Theta], x, y, 1]],
 y == 1, {x, -1, 1}, {y, -1,
 1}, {\[Theta], -\[Pi], \[Pi]}]], {t, 0, 5, 0.5}];
 Export[&quot;C:\\(*Put your directory here*).gif&quot;, fr,
 &quot;DisplayDurations&quot; -&gt; 0.3, &quot;AnimationRepetitions&quot; -&gt; 10,
 ImageSize -&gt; 800];


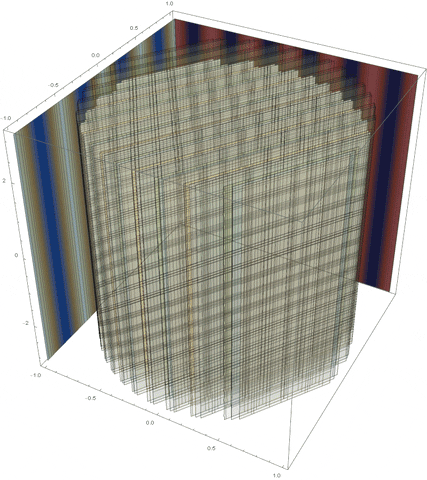


## Result 2a: Wave Equation with Slices (k1 and k2).

In this section, we examine the dynamics of the wave equation solution with respect to each of the two kime dimensions in Cartesian coordinates, where k1 and k2 range from (0,5).

In this example, we slice the 3D domain using three planes: 1) the xy-plane (the intrinsic spatial dimension), (2) the yz-plane (the dynamics corresponding to spatial dimension y and varying k2), and (3) the xz plane(the dynamics corresponding to spatial dimension x and varying k2). The time dimension k1 and k2, correspond to t and s. The k1 dimension is intrinsic in the time dimension that we perceive, and can be demonstrated by the successive pictures generated in Mathematica. The setup is

$$\xi=\left( \xi_{1},\xi_{2},\xi_{3} \right)=\left( -3,2,0 \right),\eta=\left( \eta_{1},\eta_{2} \right)=\left( -2,3 \right) .$$

eta = {-2, 3};
chsi = {-3, 2};
u[s_, x_, y_, t_] =
Exp[2*Pi*I*Inner[Times, eta, {t, s}, Plus]]*
Exp[2*Pi*I*Inner[Times, chsi, {x, y}, Plus]];
fr1 = Table[
 SliceContourPlot3D[Re[u[s, x, y, t]],
 &quot;CenterPlanes&quot;, {x, -1, 1}, {y, -1, 1}, {s, 0, 5},
 PlotLegends -&gt; Automatic], {t, 0, 5, 0.1}];
Export[&quot;C:\\(*Put your directory here*).gif&quot;, fr1,
&quot;DisplayDurations&quot; -&gt; 0.3, &quot;AnimationRepetitions&quot; -&gt; 10,
ImageSize -&gt; 800];


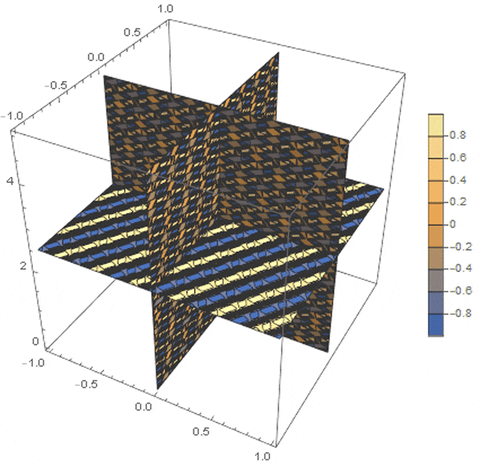


The step size here is important. For example, if we choose the step size to be 1 or 0.5, we would get a static animation result.

## Result 2b: Wave Equation with Slices (magnitude and phase).

In this section, we use the magnitude of the polar coordinate as time and let phase be in $(-\pi,\pi)$.

In this example, we follow the three cutting planes showcased in the previous section and demonstrate the wave equation solution in polar coordinates. The setup in this section will be

$$\xi=\left( \xi_{1},\xi_{2},\xi_{3} \right)=\left( -3,2,0 \right),\eta=\left( \eta_{1},\eta_{2} \right)=\left( -2,3 \right) .$$

eta = {-2, 3};
chsi = {-3, 2};
mp[\[Theta]_, x_, y_, t_] =
Exp[2*Pi*I*Inner[Times, eta, {t*Cos[\[Theta]], t*Sin[\[Theta]]}, Plus]]*
Exp[2*Pi*I*Inner[Times, chsi, {x, y}, Plus]];
fr2 = Table[
 SliceContourPlot3D[Re[mp[\[Theta], x, y, t]],
 &quot;CenterPlanes&quot;, {x, -1, 1}, {y, -1, 1}, {\[Theta], -\[Pi], \[Pi]},
 PlotLegends -&gt; Automatic], {t, 0, 5, 0.1}];
Export[&quot;C:\\(*Put your directory here*).gif&quot;, fr2,
&quot;DisplayDurations&quot; -&gt; 0.3, &quot;AnimationRepetitions&quot; -&gt; 10,
ImageSize -&gt; 800];


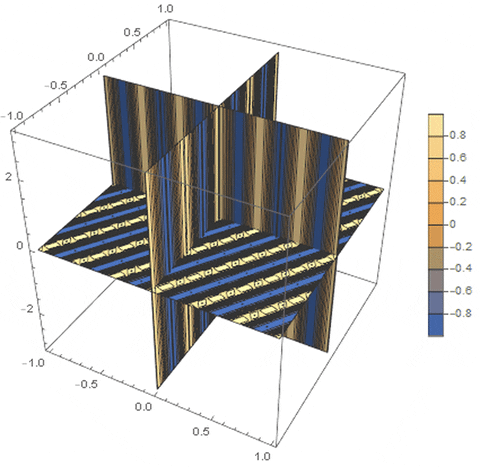


Note that the spatial dynamics in the xy-plane is similar to the Cartesian coordinates example.  To demonstrate the different phase dynamics (we only show 3 stacked planes along the x axis, as s and y are symmetric).


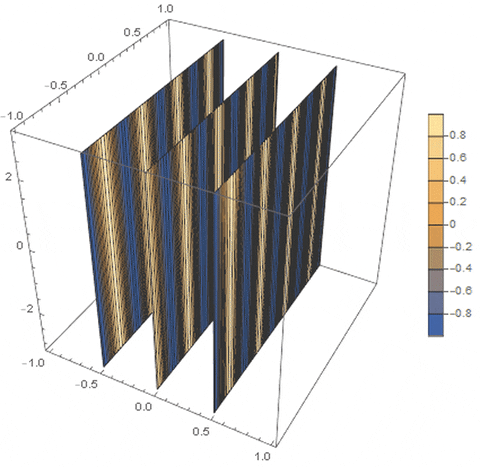


In this example, we expand the previous example to examine different spatial distributions corresponding to different phases. The output example has 9 different planar projections at different positions in the field of view.

fr2b3 = Table[Table[
 SliceContourPlot3D[Re[mp[\[Theta], x, y, t]],
 {x==l/\[Pi],y==l/\[Pi],\[Theta]==l}, {x, -1, 1}, {y, -1, 1}, {\[Theta], -\[Pi], \[Pi]},
 PlotLegends -&gt; Automatic, PlotLabel -&gt; (HoldForm["Phase"]==l) (HoldForm["Time"]==t)],
 {l,-\[Pi],\[Pi],\[Pi]/4}], {t, 0, 5, 0.1}];
 Export[&quot;C:\\(*Put your directory here*).gif&quot;, fr2b3,
 &quot;DisplayDurations&quot; -&gt; 0.3, &quot;AnimationRepetitions&quot; -&gt; 10,
 ImageSize -&gt; 800];


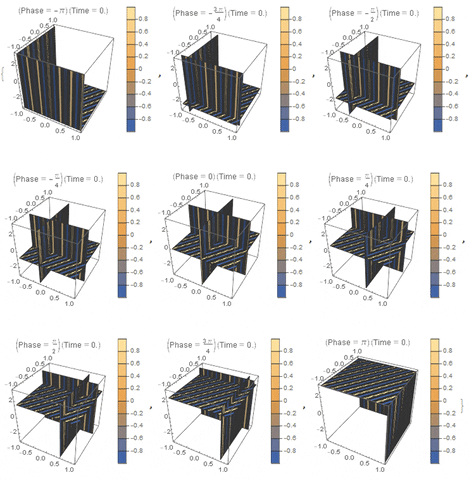


## Result 3a: Wave Equation with Spherical topology (k1 and k2).

In this section, we use the magnitude of the polar coordinate as time and limit the phase to the range $(-\pi,\pi)$.

This animation shows the wave equation solutions along spherical isosurfaces. The animation consists of 50 radial slices on the spatial coordinates, from r=0 to r=5 with 0.1 increment.

Animate[Graphics[Circle[{0,0},a],Frame -> True, PlotRange -> {{-1,1},{-1,1}}],{a,0,5,0.1},AnimationRunning -> True];

(running this code will generate animation that is displayed in the notebook)

The phase is then embedded into the polar coordinates formed by time magnitude and phase to form a spherical coordinate system. All animation iterations consist of 5 isosurfaces of the $u(x,t)$ potential function $\left( 0,1,-1,\frac{\sqrt{2}}{2},-\frac{\sqrt{2}}{2} \right)$. The segment $z<0$ and $xy<0$ is cut out to peek inside these nested isosurfaces. Also note that there is a discontinuity in the visualization as the potential function solution is not properly defined for $x=y$. The set-up is similar as in the previous section.

friso = Table[
 Show[SphericalPlot3D[{ff[\[Theta], \[Phi], r]}, {\[Theta], 0,
 Pi}, {\[Phi], 0, 2 Pi},
 Exclusions -> {\[Theta] == Pi/4},
 PlotRange -> {{-\[Pi], \[Pi]}, {-\[Pi], \[Pi]}, {-2, 2}},
 Mesh -> None,
 RegionFunction ->
 Function[{x, y, z, \[Theta], \[Phi], r}, (y > 0 || x*y > 0) ||
 z > 0], PlotStyle -> Directive[Opacity[0.5], Green]],
 SphericalPlot3D[{ff1[\[Theta], \[Phi], r]}, {\[Theta], 0,
 Pi}, {\[Phi], 0, 2*Pi},
 Exclusions -> {\[Theta] == Pi/4},
 PlotRange -> {{-\[Pi], \[Pi]}, {-\[Pi], \[Pi]}, {-2, 2}},
 Mesh -> None,
 RegionFunction ->
 Function[{x, y, z, \[Theta], \[Phi], r}, (y > 0 || x*y > 0) ||
 z > 0], PlotStyle -> Directive[Opacity[0.5], Red]],
 SphericalPlot3D[{ff2[\[Theta], \[Phi], r]}, {\[Theta], 0,
 Pi}, {\[Phi], 0, 2*Pi},
 Exclusions -> {\[Theta] == Pi/4},
 PlotRange -> {{-\[Pi], \[Pi]}, {-\[Pi], \[Pi]}, {-2, 2}},
 Mesh -> None,
 RegionFunction ->
 Function[{x, y, z, \[Theta], \[Phi], r}, (y > 0 || x*y > 0) ||
 z > 0], PlotStyle -> Directive[Opacity[1], Purple]],
 SphericalPlot3D[{ff3[\[Theta], \[Phi], r]}, {\[Theta], 0,
 Pi}, {\[Phi], 0, 2*Pi},
 Exclusions -> {\[Theta] == Pi/4},
 PlotRange -> {{-\[Pi], \[Pi]}, {-\[Pi], \[Pi]}, {-2, 2}},
 Mesh -> None,
 RegionFunction ->
 Function[{x, y, z, \[Theta], \[Phi], r}, (y > 0 || x*y > 0) ||
 z > 0], PlotStyle -> Directive[ Opacity[0.3], Blue]],
 SphericalPlot3D[{ff4[\[Theta], \[Phi], r]}, {\[Theta], 0,
 Pi}, {\[Phi], 0, 2*Pi},
 Exclusions -> {\[Theta] == Pi/4},
 PlotRange -> {{-\[Pi], \[Pi]}, {-\[Pi], \[Pi]}, {-2, 2}},
 Mesh -> None,
 RegionFunction ->
 Function[{x, y, z, \[Theta], \[Phi], r}, (y > 0 || x*y > 0) ||
 z > 0], PlotStyle ->
 Directive[Opacity[0.5], Yellow]]], {r, 0, 5, 0.1}];
 Export["C:\\(*Put your directory here*).gif", friso,
 "DisplayDurations" -> 0.3, "AnimationRepetitions" -> 20,
 ImageSize -> 800];


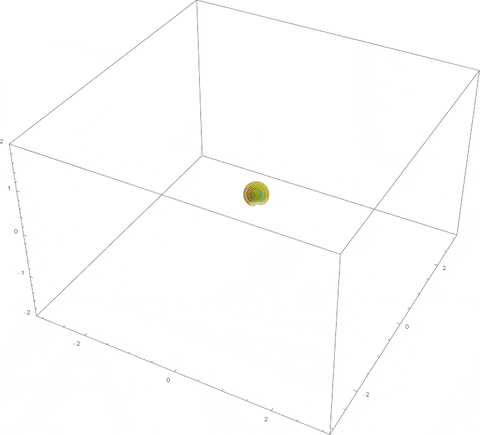


## Result 4a: Bivariate kime dynamic slider animation.

**Spatial Visualization at certain kime coordinates**

In this example, we present a dynamic slider that allows interactive manipulation along the spatial dimension at different kime coordinates (k1,k2).

*Caution*: Manual manipulation of the controls in the dynamic Mathematica app below will require some time for the Wolfram cloud server to respond. These calculations may take up to one minute to complete. Please be patient.


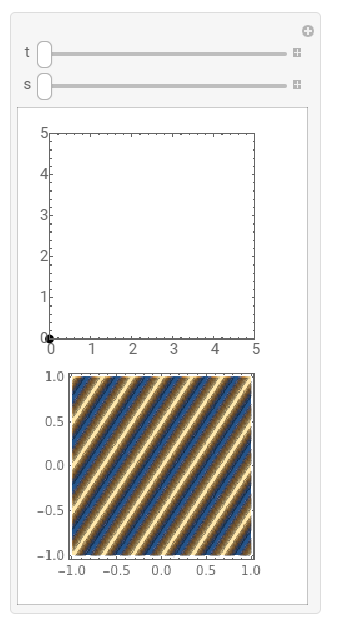


(*Be sure to run the setup in the front*)
eta = {-2, 3};
chsi = {-3, 2};
u[s_, x_, y_, t_] =
Exp[2*Pi*I*Inner[Times, eta, {t, s}, Plus]]*
Exp[2*Pi*I*Inner[Times, chsi, {x, y}, Plus]];
Manipulate[
 Column[{Graphics[{PointSize -&gt; Large, Point[{t, s}]},
 Frame -&gt; True, PlotRange -&gt; {{0, 5}, {0, 5}}],
 ContourPlot[Re@u[s, x, y, t], {x, -1, 1}, {y, -1, 1},
 Mesh -&gt; 50]}], {t, 0, 5}, {s, 0, 5}]

**Kime phases and magnitude on spatial coordinates**

In this example, we present an alternative interface that allows manipulation of the different distribution on the spatial dimension at different kime coordinates $(r,\theta)$.


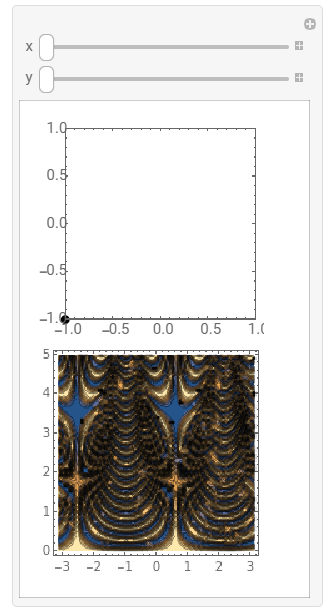


eta = {-2, 3};
chsi = {-3, 2};
int[\[Theta]_, x_, y_, t_] =
 Exp[2*Pi*I*
 Inner[Times, eta, {t*Cos[\[Theta]], t*Sin[\[Theta]]}, Plus]]*
 Exp[2*Pi*I*Inner[Times, chsi, {x, y}, Plus]];
Manipulate[
 Column[{Graphics[{PointSize -&gt; Large, Point[{x, y}]},
 Frame -&gt; True, PlotRange -&gt; {{-1, 1}, {-1, 1}}],
 ContourPlot[
 Re@int[\[Theta], x, y, t], {\[Theta], -\[Pi], \[Pi]}, {t, 0, 5},
 Mesh -&gt; 50]}], {x, -1, 1}, {y, -1, 1}]

#

# Plane Waves (Dependent kime dimensions, Path along a Closed Curve in kime space)

## Simulation: Traversing along an ellipsoid

To introduce event “orderliness” in kime coordinates, we demonstrate the dynamics of the wave equation by traversing along a closed curve in kime. The following example demonstrates the kime dependent dynamics. The problem is formulated through an observer that traverses the wave equation solution along a closed ellipsoidal path in the kime plane. The ellipsoid is defined with $a = 5, b = 3$ where we use a slightly different set up.

$$\xi=\left( \xi_{1},\xi_{2},\xi_{3} \right)=\left( -2,1,0 \right), \eta=\left( \eta_{1},\eta_{2} \right)=\left( -1,2 \right), \frac{x^{2}}{a^{2}}+\frac{y^{2}}{b^{2}}=1.$$

(*Plotting a ellipsoid*)
a = 5; b = 3;
eta = {-1, 2};
chsi = {-2, 1};
mp[\[Theta]_, x_, y_, t_] =
Exp[2*Pi*I*Inner[Times, eta, {t*Cos[\[Theta]], t*Sin[\[Theta]]}, Plus]]*
Exp[2*Pi*I*Inner[Times, chsi, {x, y}, Plus]];
frsim1 = Table[{Show[
 Plot[y /. Solve[(x/a)^2 + (y/b)^2 == 1], {x, -a, a},
 AspectRatio -&gt; Automatic, Axes -&gt; False],
 Graphics[{PointSize[Large], Blue,
 Point[{Sqrt[
 a^2*b^2/(b^2 Cos[\[Theta]]^2 + a^2 Sin[\[Theta]]^2)]*
 Cos[\[Theta]],
 Sqrt[a^2*b^2/(b^2 Cos[\[Theta]]^2 + a^2 Sin[\[Theta]]^2)]*
 Sin[\[Theta]]}]}],
 Epilog -&gt; {Text[
 StringForm[&quot;A = (``,``)&quot;,
 N[Sqrt[a^2*b^2/(b^2 Cos[\[Theta]]^2 + a^2 Sin[\[Theta]]^2)]*
 Cos[\[Theta]]],
 N[Sqrt[a^2*b^2/(b^2 Cos[\[Theta]]^2 + a^2 Sin[\[Theta]]^2)]*
 Sin[\[Theta]]]], Scaled[{.7, .7}]]}, Axis -&gt; False],
 Plot3D[Re[
 mp[\[Theta], x, y,
 Sqrt[a^2*
 b^2/(b^2 Cos[\[Theta]]^2 + a^2 Sin[\[Theta]]^2)]]], {x, -1,
 1}, {y, -1, 1}]}, {\[Theta], 0, 2 \[Pi], \[Pi]/50}];
 Export[&quot;C:\\(*Put your directory here*).gif&quot;, frsim1,
 &quot;DisplayDurations&quot; -&gt; 0.3, &quot;AnimationRepetitions&quot; -&gt; 20,
 ImageSize -&gt; 800];


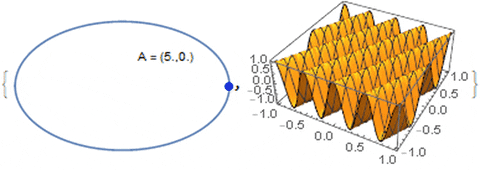


# Wave dynamics with Specified Periodic Boundary Condition

## “Heated Rod”: The unstable nature of ultrahyperbolic PDEs.

In the next example, we depict a numerical solution to an initial and boundary condition problem for the ultrahyperbolic equation (2 temporal dimensions and 2 spatial dimensions). The initial conditions are:

$$u\left( x,y,z,0 \right)=10e^{-5x^{2}-10y^{2}}, u_{t}\left( x,y,z,0 \right)=0.$$

And all the Boundary values are specified using Dirichlet boundary conditions.

eqn = Derivative[0, 0, 2, 0][u][x, y, z, t] +
 Derivative[0, 2, 0, 0][u][x, y, z, t] -
 Derivative[2, 0, 0, 0][u][x, y, z, t] == D[D[u[x, y, z, t], t], t];
(*Two initial conditions on the variable t*)
inti1 = u[x, y, z, 0] == 10*Exp[-5*x^2 - 10*y^2];
init2 =
\!\(\*SuperscriptBox[\(u\),
TagBox[
RowBox[{"(",
RowBox[{"0", ",", "0", ",", "0", ",", "1"}], ")"}],
Derivative],
MultilineFunction->None]\)[x, y, z, 0] == 0;
(*12 boundary conditions \[Equal] 3 dimensions(x,y,z) * 2 boundary \
conditions(x\[Equal]a,x\[Equal]b) * 2(first and second order)*)
bon1 = DirichletCondition[u[x, y, z, t] == 0, x == -1];
bon2 = DirichletCondition[u[x, y, z, t] == 0, x == 1];
bon3 = DirichletCondition[u[x, y, z, t] == 0, y == -1];
bon4 = DirichletCondition[u[x, y, z, t] == 0, y == 1];
bon5 = DirichletCondition[u[x, y, z, t] == 0, z == 0];
bon6 = DirichletCondition[u[x, y, z, t] == 0, z == 5];
bon7 = DirichletCondition[\!\(
\*SubscriptBox[\(\[PartialD]\), \(t\)]\(u[x, y, z, t]\)\) == 0,
 x == -1];
bon8 = DirichletCondition[\!\(
\*SubscriptBox[\(\[PartialD]\), \(t\)]\(u[x, y, z, t]\)\) == 0,
 x == 1];
bon9 = DirichletCondition[\!\(
\*SubscriptBox[\(\[PartialD]\), \(t\)]\(u[x, y, z, t]\)\) == 0,
 y == -1];
bon10 = DirichletCondition[\!\(
\*SubscriptBox[\(\[PartialD]\), \(t\)]\(u[x, y, z, t]\)\) == 0,
 y == 1];
bon11 = DirichletCondition[\!\(
\*SubscriptBox[\(\[PartialD]\), \(t\)]\(u[x, y, z, t]\)\) == 0,
 z == 0];
bon12 = DirichletCondition[\!\(
\*SubscriptBox[\(\[PartialD]\), \(t\)]\(u[x, y, z, t]\)\) == 0,
 z == 5];
usol = NDSolveValue[{eqn, inti1, init2, bon1, bon2, bon3, bon4, bon5,
 bon6, bon7, bon8, bon9, bon10, bon11, bon12},
 u, {x, -1, 1}, {y, -1, 1}, {z, 0, 5}, {t, 0, 5}];
 stabledt = {0, 0.0001, 0.001, 0.01, 0.1, 0.12, 0.13, 0.14, 0.141,
 0.142, 0.143, 0.144, 0.145, 0.146, 0.147, 0.148, 0.149, 0.15,
 0.151, 0.152, 0.153, 0.154, 0.155, 0.156, 0.157, 0.158, 0.159,
 0.16};
linearedt = {0.162, 0.163, 0.164, 0.165, 0.166, 0.167, 0.168, 0.169,
 0.17, 0.171, 0.172, 0.173, 0.174, 0.175, 0.176, 0.177, 0.178,
 0.179, 0.18, 0.181};
exponentialedt = {0.19, 0.2, 0.3, 0.4, 0.5, 1, 2, 3, 4, 5};
t11 = Table[
 DensityPlot3D[usol[x, y, z, t], {x, -1, 1}, {y, -1, 1}, {z, 0, 5},
 PlotLegends -> Automatic,
 PlotLabel ->
 Row[{Style["Stable Region", Bold, Blue], " Time: ", t,
 " s"}]], {t, stabledt}];
t12 = Table[
 DensityPlot3D[usol[x, y, z, t], {x, -1, 1}, {y, -1, 1}, {z, 0, 5},
 PlotLegends -> Automatic,
 PlotLabel ->
 Row[{Style["Center Dissipates", Bold, Red], " Time: ", t,
 " s"}]], {t, linearedt}];
t13 = Table[
 DensityPlot3D[usol[x, y, z, t], {x, -1, 1}, {y, -1, 1}, {z, 0, 5},
 PlotLegends -> Automatic,
 PlotLabel ->
 Row[{Style["Exponential Explode", Bold, Black], " Time: ", t,
 " s"}]], {t, exponentialedt}];
Export["C:\\(*.gif directory here*)",
 Join[t11, t12, t13], "DisplayDurations" -> 0.6,
 "AnimationReptitions" -> 20, ImageSize -> 800];


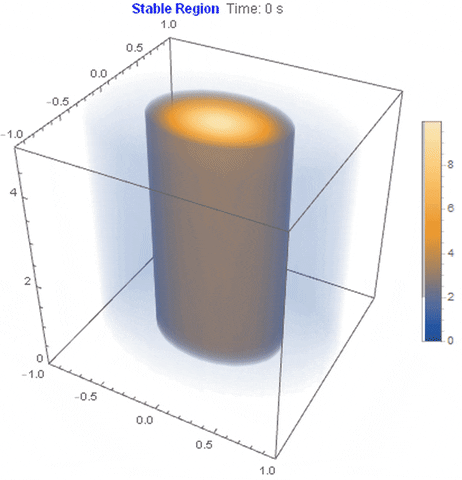


Using the same initial condition:

$$u\left( x,y,z,0 \right)=10e^{\left( -5x^{2}-10y^{2} \right)}, u_{t}\left( x,y,z,0 \right)=0.$$

And imposing the periodic boundary conditions instead.

eqn = Derivative[0, 0, 2, 0][u][x, y, z, t] +
 Derivative[0, 2, 0, 0][u][x, y, z, t] -
 Derivative[2, 0, 0, 0][u][x, y, z, t] == D[D[u[x, y, z, t], t], t];
(*Two initial conditions on the variable t*)
inti1 = u[x, y, z, 0] == 10*Exp[-5*x^2 - 10*y^2];
init2 =
\!\(\*SuperscriptBox[\(u\),
TagBox[
RowBox[{"(",
RowBox[{"0", ",", "0", ",", "0", ",", "1"}], ")"}],
Derivative],
MultilineFunction->None]\)[x, y, z, 0] == 0;
(*12 boundary conditions \[Equal] 3 dimensions(x,y,z) * 2 boundary \
conditions(x\[Equal]a,x\[Equal]b) * 2(first and second order)*)
bon1 = PeriodicBoundaryCondition[u[x, y, z, t], x == -1,
 TranslationTransform[{2, 0, 0}]];
bon2 = PeriodicBoundaryCondition[u[x, y, z, t], y == -1,
 TranslationTransform[{0, 2, 0}]];
bon3 = PeriodicBoundaryCondition[u[x, y, z, t], z == 0,
 TranslationTransform[{0, 0, 5}]];
bon4 = PeriodicBoundaryCondition[\!\(
\*SubscriptBox[\(\[PartialD]\), \(t\)]\(u[x, y, z, t]\)\), x == -1,
 TranslationTransform[{2, 0, 0}]];
bon5 = PeriodicBoundaryCondition[\!\(
\*SubscriptBox[\(\[PartialD]\), \(t\)]\(u[x, y, z, t]\)\), y == -1,
 TranslationTransform[{0, 2, 0}]];
bon6 = PeriodicBoundaryCondition[\!\(
\*SubscriptBox[\(\[PartialD]\), \(t\)]\(u[x, y, z, t]\)\), z == 0,
 TranslationTransform[{0, 0, 5}]];
usol = NDSolveValue[{eqn, inti1, init2, bon1, bon2, bon3, bon4, bon5,
 bon6}, u, {x, -1, 1}, {y, -1, 1}, {z, 0, 5}, {t, 0, 5}];


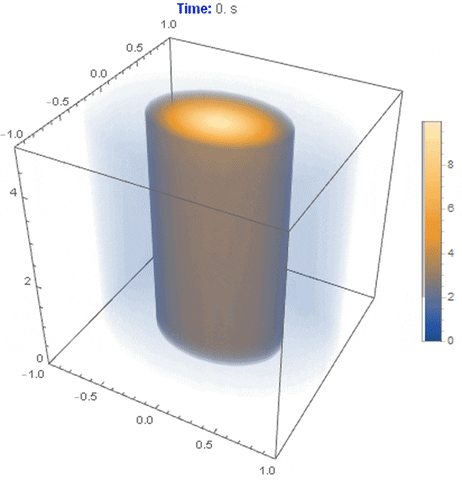


Another rendering of the solution:


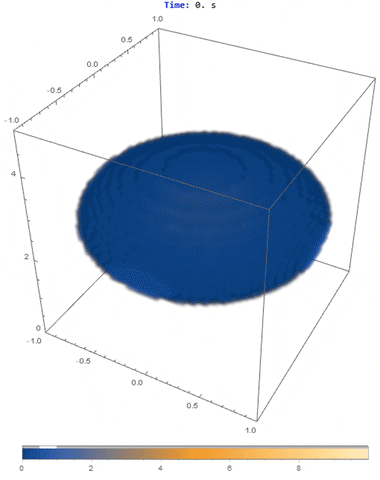


**References**:

1. Strikwerda JC. Finite difference schemes and partial differential equations: SIAM; 2004.

2. Folland GB. Introduction to partial differential equations: Princeton university press; 1995.

3. Langtangen HP. Computational partial differential equations: numerical methods and diffpack programming: Springer Berlin; 1999.

4. Quarteroni A, Quarteroni S. Numerical models for differential problems: Springer; 2009.

5. Kaiser G. Quantum Physics, Relativity, and Complex Spacetime: Towards a New Synthesis. *arXiv preprint arXiv:09100352* 2009.

6. Zhang R, Zhang Y, Liu Y, et al. Kimesurface Representation and Tensor Linear Modeling of Longitudinal Data. *Neural Computing and Applications* 2022; **in press**.

7. Dinov I, Velev M. Data Science: Time Complexity, Inferential Uncertainty, and Spacekime Analytics. 1 ed. Berlin/Boston: De Gruyter; 2021.

8. Fokas AS. A unified approach to boundary value problems: SIAM; 2008.

9. Haberman R. Elementary applied partial differential equations: Prentice Hall Englewood Cliffs, NJ; 1983.

10. Richtmyer RD, Burdorf C. Principles of advanced mathematical physics: Springer; 1978.

11. Anco SC, Gandarias ML. Symmetries, conservation laws, and generalized travelling waves for a forced Ostrovsky equation. *Partial Differential Equations in Applied Mathematics* 2022; **5**: 100230.

12. Haidar NHS. Polarization of temperature waves in a dual heat flux vector space. *Partial Differential Equations in Applied Mathematics* 2021; **4**: 100155.

13. Engler H. Similarity solutions for a class of hyperbolic integrodifferential equations. *Differential and Integral Equations* 1997; **10**(5): 815-40.

14. Kreiss H-O, Lorenz J. Initial-boundary value problems and the Navier-Stokes equations: SIAM; 2004.

15. Tolstykh VA. Partial Differential Equations: An Unhurried Introduction: Walter de Gruyter GmbH & Co KG; 2020.

16. Borthwick D. Introduction to partial differential equations: Springer; 2017.

17. Myint-U T, Debnath L. Linear Partial Differential Equations for Scientists and Engineers: Birkhäuser Boston; 2007.

18. O'Neil PV. Beginning Partial Differential Equations: Wiley; 2011.

19. Strauss WA. Partial Differential Equations: An Introduction: Wiley; 2007.

20. Morton KW, Mayers DF. Numerical solution of partial differential equations: an introduction: Cambridge university press; 2005.

21. Courant R, Hilbert D. Methods of mathematical physics, volume II. John Wiley & Sons New York; 1962.

22. Craig W, Weinstein S. On determinism and well-posedness in multiple time dimensions. *Proceedings of the Royal Society A: Mathematical, Physical and Engineering Sciences* 2009; **465**(2110): 3023-46.

23. Asgeirsson L. Uber Mittelwertgleichungen, die mehreren partiellen Differentialgleichungen 2. Ordnung zugeordnet sind (German). Studies and Essays: Interscience, New York; 1948.

24. Hörmander L. Asgeirsson's mean value theorem and related identities. *Journal of functional analysis* 2001; **184**(2): 377-401.
